# Integrative Data Analytic Framework to Enhance Cancer Precision Medicine

**DOI:** 10.1089/nsm.2020.0015

**Published:** 2021-03-18

**Authors:** Thomas Gaudelet, Noël Malod-Dognin, Nataša Pržulj

**Affiliations:** ^1^ Department of Computer Science, University College London, London, United Kingdom.; ^2^ Barcelona Supercomputing Center (BSC), Barcelona, Spain.; ^3^ ICREA, Barcelona, Spain.

**Keywords:** data integration, pan-cancer analysis, machine learning

## Abstract

With the advancement of high-throughput biotechnologies, we increasingly accumulate biomedical data about diseases, especially cancer. There is a need for computational models and methods to sift through, integrate, and extract new knowledge from the diverse available data, to improve the mechanistic understanding of diseases and patient care. To uncover molecular mechanisms and drug indications for specific cancer types, we develop an integrative framework able to harness a wide range of diverse molecular and pan-cancer data. We show that our approach outperforms the competing methods and can identify new associations. Furthermore, it captures the underlying biology predictive of drug response. Through the joint integration of data sources, our framework can also uncover links between cancer types and molecular entities for which no prior knowledge is available. Our new framework is flexible and can be easily reformulated to study any biomedical problem.

## Introduction

Over 18 million new cases of cancer and 9 million deaths were recorded worldwide in 2018.^1^ This makes cancer one of the leading causes of death. Cancer is a multi-faceted, complex disease arising from an accumulation of somatic mutations within the genome of normal cells that eventually leads to loss of normal cellular functioning and appearance of tumors that can spread across the body. Technological advances have enabled measurements from patients' tumor biopsies, including gene expression levels, DNA methylations, and somatic mutations. The research into cancer causes, and treatments, has greatly benefited from this wealth of patient data.^2,3^

Cancer projects, including The Cancer Genome Atlas (TCGA) and the International Cancer Genome Consortium (ICGC), have made publicly available wide-ranging, multi-modal, multi-omics cancer data, such as patient whole slide images, genome alterations, transcriptome, and epigenome.^4,5^ Free access to these large-scale, diverse databases has dramatically facilitated studies of the biological mechanisms of specific cancer types.^4,6,7^ The available data have also enabled pan-cancer analyses that study cancer in general to identify common mechanisms and differences across cancer types.^7,8^ Recently, the Pan-Cancer Analysis of Whole Genome (PCAWG) project^7^ has informed that our knowledge about cancer is far from complete, as 5% of their cohort was without any known cancer driver mutations. Importantly, these large databases have paved the way for the field of Precision Medicine, whose overarching aim is to improve medical care for patients by tailoring treatment to their individual molecular profiles.^9^ Precision medicine has diverse intermediary objectives, for instance, uncovering diagnostic and prognostic biomarkers. This is especially relevant to a heterogeneous disease, such as cancer, which manifests uniquely in every patient.

Complex diseases, such as cancer, can be caused by combinations of genetic, molecular, environmental, and lifestyle factors. Any single type of biological data cannot fully capture such diseases. As such, collective mining of different data has been gaining momentum as a means to extract integrated system knowledge that goes beyond what any single data source can offer.^10^ This principle applied to the study of cancer has enabled the discovery of cancer-related genes, or group of genes,^11–13^ and the identification of cancer subtypes significantly correlated with patient prognoses.^12,14^

Biological data often have a small number of samples relative to the number of available features. For instance, a typical dataset in TCGA contains a few 100 patients who are each characterized by tens of thousands of features (e.g., expression levels of around 20,000 genes). However, biological features are often redundant due to underlying molecular interactions among biological entities.^15^ This has been a motivation for the use of dimensionality reduction and embedding algorithms that are pervasive in bioinformatics.^16^ In addition, due to the low sample to features ratio, dimensionality reduction techniques are often necessary as data pre-processing for machine learning models.^16^

Non-negative matrix factorization (NMF) approaches are unsupervised algorithms that have extensively been used both as a means to integrate heterogeneous data and reduce data dimensionality. They encompass all methods that decompose a matrix, representing relational links between two sets of entities, into the product of low-dimensional, latent, positive matrices, or factors, whose sizes control the degree of dimensionality reduction.^17^ Importantly, they can be used to derive an embedding in an unspecified latent space for each entity. Matrix factorization approaches have had numerous applications, including collaborative filtering^18^ and biological data integration for cancer analysis.^12,14^ Reconstructing a matrix based on a factorization has often been used to make predictions and infer new knowledge.^12^ NMF approaches have been successfully applied as pre-processing steps for downstream machine learning classifiers.^19^

**FIG. 1.**
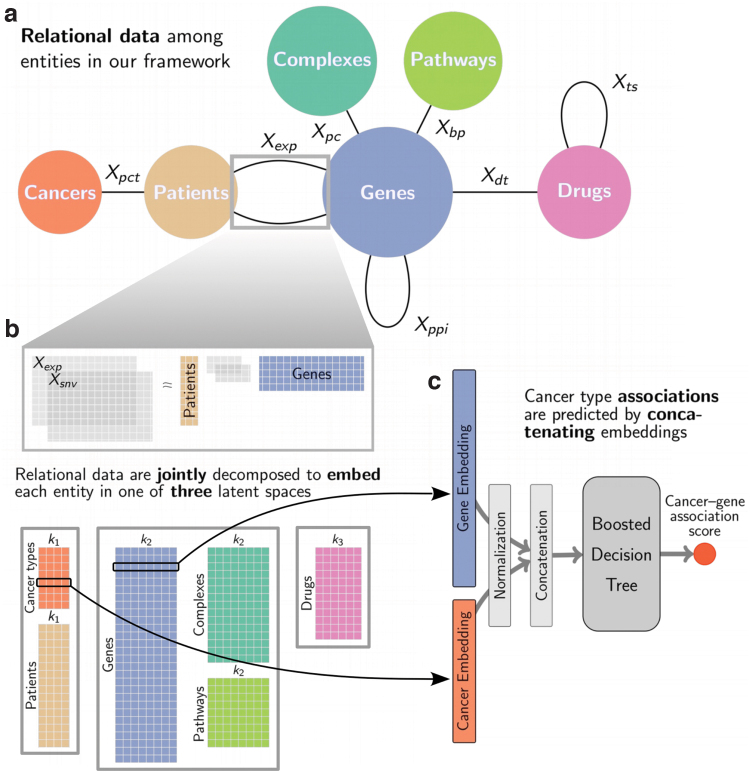
**(a)** Input to our matrix factorization embedding model: relational data between entities. Each edge corresponds to a type of link and to a subobjective of our joint factorization model (see Supplementary Table S2 for notations). The squares group entities that are embedded in the same joint latent space. **(b)** Illustration of the NMTF factorization subobjectives corresponding to the edges in the gray box in **(a)**. Each group of entities is associated in the decomposition to a factor that is shared across all subobjectives involving that group of entities. Through the joint decomposition of all relational data, we derive embeddings for each entity in three latent spaces, indicated by the gray boxes, with dimensions *k*_1_, *k*_2_, and *k*_3_. **(c)** We predict associations relevant to cancer types with boosted decision trees taking as input, for instance, the concatenation of the embeddings of a cancer type and of a gene and predicting if the two are linked. NMTF, non-negative matrix tri-factorization.

We propose a pan-cancer framework to uncover cancer type-specific molecular mechanisms and identify drugs that could be repurposed (Fig. 1). Our framework relies on the simultaneous integration and dimensionality reduction of various data using a joint NMF model. Our framework includes more data than the previous studies, integrating patient-specific diagnosis, gene expression, and single-nucleotide variants, (SNV) as well as generic network data on human: protein–protein interactions, protein complex associations, biological pathways, drug–target interactions, and drug chemical similarities. To integrate the wealth of data in one framework, we rely on three types of matrix factorizations: NMF, non-negative matrix tri-factorization (NMTF), and symmetric non-negative matrix tri-factorization (SNMTF). The details for each can be found in Supplementary Methods section. Data integration is achieved by jointly optimizing for multiple factorization objectives with shared factors (see Supplementary Methods section). We obtain a *context-aware* embedding of each entity (cancer type, patient, gene, complex, pathway, and drug) that takes into account all the input data.

Using boosted decision trees, we predict biologically relevant associations between cancer types and genes, drugs, pathways, and complexes based on the context-rich embeddings of our entities. One key insight is that the integration step, by construction, embeds the entities into three latent spaces, each associated with a different family of entities: (1) patient-related entities (i.e., patients and cancer types), (2) gene-related entities (i.e., genes, complexes, and pathways), and (3) drugs. This means that the entities in a given latent space can be substituted with each other when using a model trained to predict associations between one of these classes of entities and cancer types. In this respect, our approach is similar to zero-shot learning,^20^ which aims to accurately classify at test time samples that belong to classes unseen at training time. In our case, we aim to predict the association of cancer types to unseen classes of entities at training time. Finally, our approach is able to predict patient's response to drugs, implying that our framework captures important biology that governs response to cancer drugs.

## Materials and Methods

### Our framework for context-aware embeddings

The core of the framework is gene information (Fig. 1a). We integrate three types of data about genes. We obtain RNA sequencing data and SNV data for 7998 patients from ICGC (see Supplementary Methods section for details) across 21 cancers. Henceforth, we refer to each cancer by its abbreviation given in Supplementary Table S1. We obtain data on gene interactions, including protein–protein interaction (PPI) network from Biogrid, protein complexes from Reactome and Corum, and biological pathways from Reactome (see Supplementary Methods section for details). These data capture physical and functional relationships between genes and are used to anchor our framework within the context of molecular interactions. The last type of gene data corresponds to drug–target interactions from DrugBank (see Supplementary Methods section for details), connecting drugs to proteins that they target. We further add drug chemical similarity information to push similar drugs closer in the latent space. We also add patient diagnosis information through which we embed cancer types and patients in a joint latent space to both push patients closer if they have the same cancer and push molecularly similar cancer types closer. This could help tailor treatments to patients by placing them within a cancer “space” since cancer is a heterogeneous disease and a given cancer type might manifest differently in different people. This may aid characterizing cancer of each patient as accurately as possible to personalize treatment options.

Because of the heterogeneity of our input data, our integration framework is based on joint optimization of different variants of NMF: classical NMF, NMTF, and SNMTF. Each variant, described in Supplementary Methods section, is best fitted for the decomposition of a different type of relational data. In particular, we use SNMTF to factories the PPI network and the drug similarities matrix, NMTF to factorize patient molecular data and drug–target data, and NMF for the remaining data. Each edge in Figure 1a corresponds to a subobjective of our embedding framework, that is, a specific NMF decomposition. In the joint decomposition, each group of entities is associated with a factor that is shared across all subobjectives involving that group of entities. For instance, the patient factor is shared by all subobjectives that involves patient-specific data (diagnoses, gene expressions, and somatic mutations). An entity's embedding is obtained from the factor of the associated group of entities.

Through our integrative framework, we derive embeddings for all entities (cancer types, patients, genes, pathways, complexes, and drugs) that best fit the full context of the framework, that is, the input relational data. Each entity's embedding, in one of the three latent spaces learnt by our framework, encapsulates the information from the input data that is relevant to that entity; thus, we say that this representation is *context aware*. Our framework has three hyperparameters, denoted by *k*_1_, *k*_2_, and *k*_3_, which correspond to the dimensionalities of the latent spaces. To find suitable values for these hyperparameters, we perform a grid search with $k_{1}\in \left\{2,5,10,15,21\right\}$, $k_{2}\in \left\{70,80,90,100,110\right\}$, and $k_{3}\in \left\{40,50,60,70,80\right\}$. The former is a coarse grid over the range of possible values. For the latter two, due to the large range of possible values, the intervals are restricted around the value $\sqrt{n∕2}$, where *n* is either the number of genes or the number of drugs. $\sqrt{n∕2}$ corresponds to a heuristic commonly used to set the number of clusters.^21^

As the selection criterion, we measure if each patient tends to be embedded in the latent space closer to their diagnosis than to other cancer types. We quantify this with the macro-F1 score of the classifier that associates to each patient the closest cancer type in the latent space in terms of cosine distance. We found that the following hyperparameters values maximize this metric: $k_{1}=21$, $k_{2}=70$, and $k_{3}=40$. Supplementary Figure S1 shows the sensitivity of different metrics to the choice of the hyperparameters, which we discuss in the rest of the article.

Each iteration of our integrative model takes around 10 seconds, depending on hyperparameters, on 8 CPUs (Intel Xeon E5-2650 v3 @ 2.30 GHz) with 16G of RAM. With *n* denoting the total number of entities, our matrix factorization-based framework has time complexity $O\left(n^{3}\right)$ and memory complexity $O\left(n^{2}\right)$.

### Predicting cancer type associations

To extract new knowledge for each cancer type, we use our context-aware embeddings to suggest cancer–drug and cancer–gene associations. We cast the problem as a link prediction task for which we train boosted decision trees to predict known associations from our entities' embeddings. After our training step, we use the trained classifiers to predict new associations (see Supplementary Methods section). As pre-processing, we normalize all embeddings to have a unit norm. The normalization step is crucial for the transfer of a link predictor from one type of entity to another that we discuss in the next section. For each possible cancer–drug pair (or cancer–gene pair), we define the pair's representation by the concatenation of the embeddings of its components, that is, the concatenation of the cancer's embedding vector with the drug's embedding vector defines the feature vector of the pair. Finally, we use boosted decision trees for link prediction, taking as input a pair's representation and output the association's scores of its component (Fig. 1c). We choose boosted decision trees due to their simplicity and high performances in a number of competitions.^22^

In the first validation step, we systematically evaluate the performance of our approach with a 10-fold cross-validation using both the area under the receiver operating characteristic (AUROC) and the area under the precision recall curve (AUPRC) and compare our results to state-of-the-art methods for link prediction. The two metrics are often used in concert as they characterize different aspects of the results. Notably, AUPRC gives robust evaluations in imbalanced settings. The splits used for the 10-fold cross-validation are performed on the set of known links and considering all nonreported links as part of the negative set of links. Note that we do not use any balancing strategy during training. Furthermore, we perform an ablation study on patient–gene data (Supplementary Fig. S6), that is, we compare the results obtained with those obtained with framework using less patient data to demonstrate the interest of considering both expression and mutation data jointly.

In the second step, we investigate the top 10 drugs and genes associated with cancer types by our methodology. Each pair is scored based on the average of the standardized scores given by 10 classifiers trained for the cross-validation (see Supplementary Methods section for details). In this step, we only consider drugs and genes that were thus far not associated with any cancer type in the ground-truth data (introduced in each subsection) to avoid trivial cases of information transfer from one cancer to another, which typically happens when one drug or one gene is associated with a majority of cancers. We perform a manual literature curation to validate the top results.

## Results and Discussion

### Patient and cancer embeddings are medically relevant

**FIG. 2.**
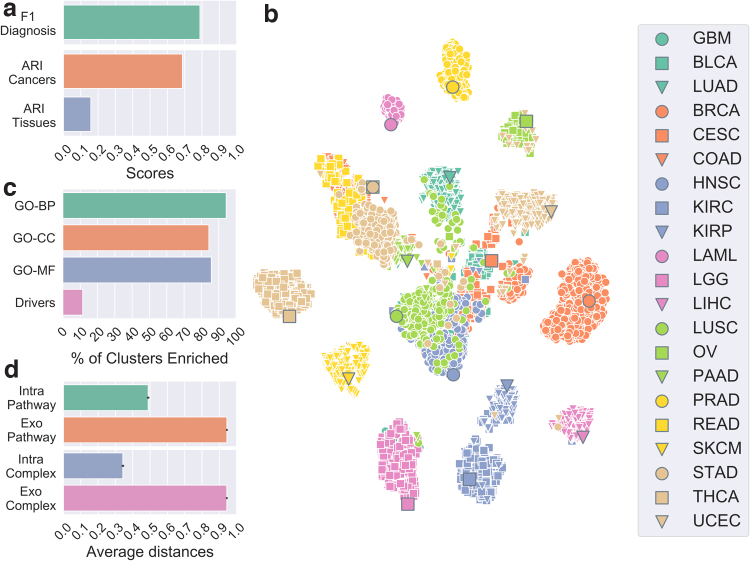
**(a)** Macro-F1 score quantifying the relationship between a patient and its cancer type (green) and ARI measuring the link between patient clustering for each model and cancer type labeling (orange) and tissue-sampled labeling (blue). **(b)** t-SNE plot representing the embedding of patients and cancer types in the latent space. The larger circled markers correspond to the embeddings of cancer types, and the smaller ones represent the embeddings of patients. Colors and shapes indicate cancer types (see Supplementary Table S1 for abbreviations meanings). **(c)** Percentages of gene clusters enriched in GO-BP, GO-CC, GO-MF, and driver annotations. **(d)** Average cosine distance between genes and associated pathways and complexes (intrapathway and intracomplex) and nonassociated pathways and complexes (exo-pathway and exo-complex). ARI, adjusted rand index; BP, biological processes; CC, cellular component; GO, Gene Ontology; MF, molecular function; t-SNE, T-distributed stochastic neighbor embedding.

To evaluate the biomedical relevance of our joint patient and cancer embeddings, we observe that the macro-F1 score is close to 0.8 for our optimal set of hyperparameters (Fig. 2a), indicating that the majority of patients are embedded closer to their diagnoses than to other cancer types. In addition, we evaluate if patients group in the latent space with respect to either cancer type or a sampled tissue. To this end, we use hierarchical clustering with cosine distance to group patients in *k* groups (where *k* is either the number of cancers or the number of tissues) and compute the adjusted rand index (ARI) to measure the link between the clustering and the ground truth labeling (either cancer types or sampled tissues; see Fig. 2b). We observe that patients do not cluster well with respect to sampled tissues, having ARI below 0.2. However, we observe ARI 0.7 with respect to cancer type, indicating that our clusterings resemble diagnostic labeling with some discrepancies. These results are expected, as the inclusion of patient diagnosis data in the framework implies a constraint that aims to embed each patient close to their diagnosis and subsequently to other patients having the same disease. Note, however, that some patients do not fit well with the rest of their cohorts. This is an important observation, as it suggests that those patients might need different care options from the majority of their cohort and further motivates personalizing treatments to individual patients.

As an illustration, we visualize our latent space embeddings using T-distributed stochastic neighbor embedding (t-SNE). t-SNE is a machine learning algorithm for nonlinear dimensionality reduction, well suited for visualization in a two-dimensional space of high-dimensional data.^23^ We observe as expected that patients tend to cluster according to cancer type, with the cancer type itself being also embedded nearby (Fig. 2c). In addition, we observe that some cancers are grouped in a meaningful way. For instance, both brain cancers, glioblastoma multiforme and lower grade glioma, form one group. The cluster in the center contains mostly squamous cell carcinomas, head and neck squamous cell carcinoma, cervical squamous cell carcinoma, and lung squamous cell carcinoma (LUSC). Both cancers that affect kidneys, kidney renal papillary cell carcinoma and kidney renal clear cell carcinoma, are also grouped. Moreover, rectum adenocarcinoma, colon adenocarcinoma, and stomach adenocarcinoma—which are cancers affecting rectum, colon, and stomach, respectively—form another cluster. We also observe that some patients having a specific type of cancer do not group with the majority of the cohort. Note that the same observations hold when using Uniform Manifold Approximation and Projection algorithm^24^ instead of t-SNE (Supplementary Fig. S7).

We further investigate if our framework learns a meaningful latent space that translates into actionable representations for new unseen patients. We perform a 10-fold cross-validation in which 90% of patients of each cancer type are used to derive the embeddings of all entities in the framework. We then project the remaining 10% of patients in the derived cancer/patient latent space (see Supplementary Methods section). First, we test if these patients are placed close in space to their diagnosis (quantified as above, see Supplementary Table S5). This gives a macro-F1 score of 0.77±0.03, which is close to the score obtained with all patients included in the framework (∼0.8). This shows that new patients are placed in the latent space according to their diagnoses with accuracy similar to that for patients included in the framework.

We also test if the unseen patients tend to be embedded closer to patients having the same diagnoses. For this, we use a *k*-nearest neighbor classifier with k=10 (see Supplementary Methods section) and measure its macro-F1 score (Supplementary Table S1). We observe a score of 0.88±0.02, which shows that the large majority of new patients are embedded in the latent space closer to patients diagnosed with the same type of cancer. Both results show that our latent space is robust in the sense that we can derive an embedding for new patients that is consistent with that of known patients and cancer types. We also observe that the *k*-nearest neighbor algorithm gives a more robust diagnosis classifier than finding the closest cancer in the latent space. This means that the local neighborhood of a patient in the latent space is a better diagnosis indicator than a global predictor derived from cancer types' embedding. This suggests the presence of patient subgroups within a cancer type that display substantially different molecular behavior.

Overall, our analysis shows that the patients/cancers latent space is consistent with known biology. Furthermore, our framework has the advantage of relaxing the hard clustering derived from patients diagnoses through patient's molecular similarity, highlighting that a patient's molecular profile can be more similar to the profiles of patients with different cancers than to the profiles of patients with the same diagnosis. This observation motivates further the need for pan-cancer perspectives in precision medicine.

### Our gene latent space is biologically relevant

To evaluate the biological relevance of our genes embeddings, we cluster them in *k*_2_ group using cosine distance-based hierarchical clustering, and measure the enrichments of the clusters in terms of Gene Ontology (GO) annotations and in terms of cancer driver genes (see Supplementary Methods section for details, Supplementary Table S3). We consider all three subtypes of GO annotations: Biological Processes (GO-BP), Cellular Component (GO-CC), and Molecular Function (GO-MF), separately. The significance of the enrichments is computed with a hypergeometric test with Benjamini-Hochberg correction for multiple hypothesis testing and a significance threshold of 0.05. We observe that, regardless of the GO subtype, above 80% of clusters are significantly enriched in at least one annotation (Fig. 2c and Supplementary Fig. S2 for cluster size and number of enriched annotations).

We further evaluate the meaningfulness of our results by performing 10,000 randomized tests where clusters' sizes are held constant, but genes are randomly assigned. We obtain 0% of clusters enriched with random cluster assignment at least 9650 times out of 10,000 repeats (empirical *p*-values 0.035) for each type of annotation. These results show that genes with similar function are embedded closer in the latent space and thus our genes' embeddings significantly capture known biology. Interestingly, we also observe that around 10% of the clusters are enriched in cancer driver genes, indicating that cancer drivers are embedded closely, that is, clustered, in the latent space. This highlights the link between the gene latent space and the cancer context that we made a part of our framework. Furthermore, it underlines the relevance of our embeddings for the identification of putative cancer-related genes, discussed in the following section.

In addition, we perform an ablation study on the gene interaction data input to investigate the effect that each dataset has on enrichment scores (Supplementary Fig. S3). For each model, all hyperparameters are selected following the same procedure outlined above. First, we observe that adding any gene data is better than not adding them from the point of biological annotation enrichment. For instance, the model without any PPI, complex, or pathway data has 40% of gene clusters enriched in GO-BP annotations, while every model with at least one data source has above 80% of gene clusters enriched for the same annotations. However, there is no clear best model among the ones with diverse combinations of the data, each scoring similar enrichment values with different models performing slightly better for different annotations. Thus, different combinations of data do not seem to lead to significantly different performances, but keeping all data in the model enables analysis of each class of entities.

Finally, as pathways and complexes are embedded in the same latent space, we investigate their positioning with respect to genes. In particular, we evaluate if a gene is embedded closer to its associated higher-order entities, that is, pathways and complexes, than to those to which it has not been associated yet. To this end, we compute the cosine distances in the latent space between a gene and its associated pathways and complexes, termed “intrapathway” and “intracomplex” distances, as well as the distances between the gene and all nonassociated pathways and complexes, termed “exo-pathway” and “exo-complex” distances. We observe that genes are embedded closer, on average, to their associated higher-order entities than they are to those that they are not associated to (Fig. 2d), with average distance below 0.5 between a gene and associated entities and above 0.9 between a gene and nonassociated entities. These results are significant according to a Mann–Whitney *U* statistical test (*p*-value ∼0 in both cases) and underline the relevance of the joint embedding of genes with related higher-order molecular structures in the same latent space. This also suggests that our framework could be used for identifying new genes that are involved in or interact with pathways and protein complexes, which we leave for future work.

### Cancer type associations

#### Our model predicts relevant treatment

To predict cancer–drug associations, we train decision trees to identify known associations that we collect from DrugCentral^25^ (last updated October 2018). DrugCentral contains 93 associations in total between our sets of cancer types and drugs. We define our positive set with DrugCentral treatment options and consider all nonreported associations for our negative set. Our classifier takes as input the concatenation of the normalized embeddings of a drug and a cancer type and outputs their association score.

We compare our results to four baseline methods: non-negative matrix factorization reconstruction (NMFR), measure-based bidirectional random walks (MBiRW),^26^ Drug Repositioning Recommendation System (DRRS),^27^ and bounded nuclear norm regularization (BNNR)^28^ (see Supplementary Methods section for implementation details). These methods rely on the assumption that similar diseases share treatment, and conversely, that similar drugs can treat the same disease. MBiRW uses random walks to derive repurposing hypothesis by discovering neighborhoods in similarity networks. DRRS and BNNR embed drugs and diseases in low-dimensional latent spaces with the underlying hypothesis being that missing links emerge from those latent spaces using the inner product. Our method makes similar assumptions, but takes more information into consideration, notably drug targets as well as protein interactions. Furthermore, by using boosted decision trees on the latent spaces, our model gains more flexibility and representative power over the simple inner product.

**FIG. 3.**
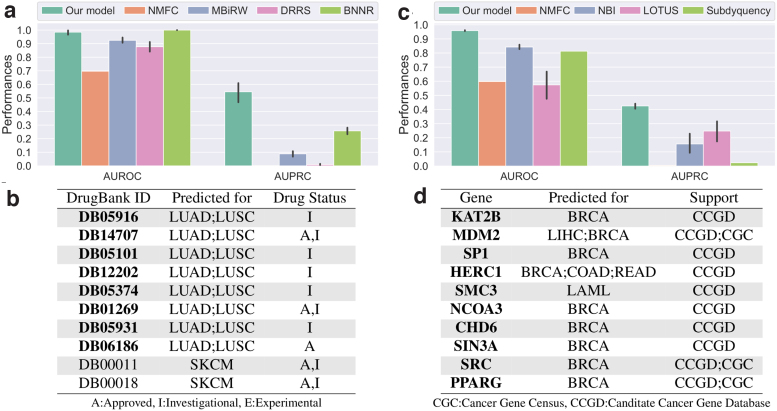
Performances of our cancer–drug association predictor (left column) and cancer–gene association predictor (right column). The bar charts give the performances of our classifiers measured with 10-fold cross-validation in terms of AUROC and AUPRC. The tables give the top 10 associations between cancers and drugs **(b)** and genes **(d)** that are not associated with any cancer in our data. Drugs or genes highlighted in bold font have been associated to cancer. The support column in the bottom right table indicates which database lists a link between the gene and cancer. AUPRC, area under the precision recall curve; AUROC, area under the receiver operating characteristic.

We observe that our approach significantly outperforms the competing methods (Fig. 3a). BNNR achieves slightly better AUROC scores (∼0.99 compared to our ∼0.97), but it scores significantly lower than our framework in terms of AUPRC (∼0.25 compared to our ∼0.5). These results show the relevance of our method when compared to the state-of-the-art drug repurposing approaches. We analyze further the results of our approach through literature curation for the top-scoring drugs that are not associated with any cancer types in DrugCentral.

Among the top 10 drugs that are the most associated to cancer types by our classifiers (Fig. 3b), a majority is recorded in DrugBank as investigational or approved for the treatment of some cancers. The approved predicted drugs either are not present in DrugCentral, as their approval postdates the DrugCentral release, or target a cancer type not considered in this study. We discuss below supporting information for our top 3 predicted drugs. We provide validations of all of our predictions in the Supplementary Data.

DB05916 (CT-011) targets gene *PDCD1*, which has immunomodulating and antitumor activities. CT-011 is currently being investigated for the treatment of tumors and unspecified cancers.^29^ DB14707 (Cemiplimab) is an FDA-approved drug for the treatment of advanced cutaneous squamous cell carcinoma.^29^ Our classifier suggests that it could be used to treat lung cancer and notably LUSC. DB05101 (Matuzumab) is an investigational drug that targets *EGFR* gene, which is often associated with cancers, including lung cancers.^30^

The manual literature curation highlights that our predicted drugs are often investigated or approved for the treatment of forms of cancer and that their targets, or mechanisms of actions, can be linked to the specific cancer types we predict. Overall, the analysis strongly supports our methodology.

#### Our framework identifies genes relevant to cancer types

Based on known cancer genes from IntOGen,^31^ we train classifiers to identify associations between genes and cancer types. In total, IntOGen reports 1129 associations between our sets of cancer types and genes. These interactions constitute our positives set. All nonreported associations are considered part of our negatives set. As above, a decision tree takes as input the concatenation of the normalized embeddings of a gene and a cancer type and outputs their association score.

We compare the performance of our method with the following state-of-the-art methods: NMFR, network-based integration (NBI),^32^ LOTUS,^33^ and Subdyquency.^34^ The methods were developed to predict cancer-related genes in slightly different contexts and are adapted to our problem here (see Supplementary Methods section for details). The baselines rely on the “guilt-by-associations” where genes that are linked to a disease, but are not necessarily mutated or differentially expressed, can be identified in biological networks through links to known disease genes. NBI and Subdyquency use random walks to exploit proximities on networks to identify such genes. LOTUS uses kernels to capture similarities between cancers and between genes, combining the two kernels to feed to a Support Vector Machine algorithm. Our approach draws from similar ideas, combining both factorization to augment entity proximities through latent space embedding and machine learning model. Furthermore, our approach can integrate additional protein interaction data, notably functional protein interaction (pathways).

We observe that our approach outperforms the competing methods (Fig. 3c) in terms of AUROC, which is over 0.9 for our method compared to below 0.8 for the other approaches, and in terms of AUPRC, which are around 0.4 for our approach compared to below 0.25 for the other methods. We further evaluate if our approach accurately captures known cancer-related genes that are reported in Candidate Cancer Gene Database (CCGD), but not in IntOGen. We perform this analysis both globally and on a per cancer basis (note that 14 cancer types have data for this test). We observe that our method ranks highly associations between genes and cancer types in CCGD, notably giving AUROC scores above 0.59 (*p*-values $<10^{-7}$) for all cases (Supplementary Fig. S4). Below we look at the top 10 genes that are identified by our method (Fig. 3d). We use the Cancer Gene Census (CGC)^35^ and CCGD^36^ to find known associations, as well as literature curation (both databases were accessed in August 2019).

We observe that our top 10 scoring genes are listed in either the CCGD or the Cancer Gene Census (CGC) as linked to at least one form of cancer. Furthermore, the pairs *MDM2*–LIHC, *HERC1*–COAD, *HERC1*–READ, *SMC3*–LAML, *NCOA3*–BRCA, and *CHD6*–BRCA are associated in CCGD. In addition, *KAT2B* (*PCAF*) activity has been linked to cancers, and in particular, to breast cancers, in the literature.^37–39^ *MDM2* has also been associated with breast cancer.^40^ *SP1* expression has been linked to breast cancer in multiple prior studies.^41–43^

For each of these three genes, we stratify our breast invasive carcinoma (BRCA) cohort into two groups: patients having higher than average expression of the gene and patients having lower than average expression of the gene. We compute a logrank statistical test (with 0.05 cutoff) and observe that for each of the three genes, the patient groups have significantly different survival rates with *p*-values 0.002 for *KAT2B*, 0.026 for *MDM2*, and 0.039 for *SP1* (see Supplementary Fig. S5a–c for Kaplan-Meir plots). For each of the three genes, higher expressions are associated with lower survival rates. We provide validations for the remaining predictions in the Supplementary Data. Note that BRCA is overrepresented in the results. However, the global validation results (Supplementary Fig. S4) do not indicate a bias toward BRCA and a possible explanation for this observation is that BRCA is associated with more driver genes than the other cancer types in this study (96 driver genes compared to no more than 74 for other cancer types).

The literature curation highlights that each gene identified through our approach is relevant to the associated cancer type, with support through existing research and databases, as well as statistical evidence for a connection between gene expression level and patient prognosis. Thus, this supports our methodology.

### Repurposing boosted decision trees

Links between types of entities are not always known or available (e.g., associations between cancer type and protein complexes or associations between patients and drugs), which prevents us from using the same methodology to derive new knowledge. However, our framework allows for extrapolating those links from known associations between other types of entities. Our approach relies on the previously observed fact that, by design, some entities are embedded in the same latent spaces (e.g., genes, pathways, and complexes or cancer types and diseases). We have further shown, in the first section, that the relative location in the latent space of related entities was biologically consistent, that is, related entities are closer to each other than nonrelated entities. Based on these observations, we postulate that a classifier trained from the embeddings of a given type of entities can be repurposed to predict from the embeddings of another type of entities. For instance, decision trees that learnt to associate genes to cancer types can be used to predict which biological pathways or protein complexes could be associated with which cancer types. This could effectively provide insights into the impact of cancers onto cells by identifying affected higher-order cellular structures and functions. We focus below on the analysis of top associations between biological pathways and cancer types. Similar results can be obtained for protein complexes and are discussed in Supplementary Results section and Supplementary Table S6.

The association score between a cancer type and a biological pathway is obtained by simply feeding the concatenation of the normalized embeddings of both entities to the 10 decision trees trained to predict cancer–gene associations. The average of the standardized scores across all decision trees gives the final association score.

Comparative Toxicogenomics Database (CTD) database^44^ gives associations between diseases and pathways based on shared associated genes and can be used for global validation. We achieve an AUROC score of 0.65±0.01 and an AUPRC score of 0.66±0.01, which indicate predictions significantly better than random (*p*-value ∼0) for our repurposed decision trees. However, note that 52% of all possible associations between our set of cancer types and our set of pathways are reported in the database. This indicates that the condition for association used by CTD might not be sufficiently stringent. This motivates the following manual literature curation to validate our top 10 scoring biological pathways (see Table 1). We discuss the first three predicted pathways below and provide validations of all remaining predictions in the Supplementary Data.

**Table 1.** Top 10 biological pathways associated to cancer types

Pathway	Predicted for
R-HSA-112411	All cancers
R-HSA-2262752	BRCA
R-HSA-5654688	BRCA; GBM; BLCA; LGG; PAAD; LAML; LUSC; STAD; SKCM; LUAD; UCEC
R-HSA-5654699	BRCA
R-HSA-8953897	BRCA
R-HSA-110056	BRCA
R-HSA-389357	BRCA
R-HSA-5654719	BRCA
R-HSA-9603381	BRCA
R-HSA-8866910	BRCA

BLCA, bladder urothelial carcinoma; BRCA, breast invasive carcinoma; GBM, glioblastoma multiforme; LAML, acute myeloid leukemia; LGG, low grade glioma; LUSC, lung squamous cell carcinoma; SKCM, skin cutaneous melanoma; STAD, stomach adenocarcinoma; UCEC, uterine corpus endometrial carcinoma.

*MAPK1 (ERK2)* activation pathway (R-HSA-112411) and *MAPK3 (ERK1)* activation pathway (R-HSA-110056) have been linked to numerous cancers, such as breast cancer, as discussed in the previous section, and colorectal cancer.^45^ The *ERK MAPK* pathway is critical for cell proliferation and thus is naturally often connected to cancers. Cellular responses to stress pathway (R-HSA-2262752) is a subpathway of the cellular responses to external stimuli pathway (R-HSA-8953897).^46^ Anticancer treatments are often successful when able to induce apoptosis through external stimuli that induce cellular stress.^47^ For instance, tumor suppressor gene *P53* can be stimulated through cellular stress.^48^ Thus, perturbation to those pathways might lead to cancer onset and resilience to treatment.

The literature review highlights the ability of our repurposing approach to identify associations between biological pathways and cancer types that are supported by the existing literature. Thus, this analysis underlines the ability of our framework to extract biological pathways associated with cancer.

### Predicting patients' responses to cancer drugs

We collect data on patient responses to cancer drugs from TCGA.^4^ We only consider patients and drugs that are present in our dataset. The task corresponds to a binary classification where we predict if a patient's response to a drug is positive or negative. A response is considered positive if TCGA reports a complete response of the patient, and negative otherwise. As we are interested in finding drugs that improve the state rather than maintain a status quo, or degrade the state, we believe it is reasonable to use a binary variable for this task. We further discard 100 entries corresponding to combinations of drugs as our model is not suitable for the analysis of these data. From the remaining data, we only consider drugs that have both positive and negative response. After processing, we have 2589 patient–drug pairs. We split these data in train, validation, and test sets with a 70%/10%/20% partition, repeating the experiment 10 times and measuring AUROC and AUPRC scores.

We train a boosted decision tree model to predict patients' responses to drugs. As above, the input to the model is the concatenation of the normalized embedding of a patient and a drug. The output can be interpreted as the success probability of the treatment. Our approach performs well, achieving AUROC score of 0.869±0.013 and AUPRC score of 0.855±0.014. This result suggests that our model is able to capture some common biological mechanisms that govern response to cancer drugs.

To analyze this claim further, we investigate which features the models use most to predict response. First, we compute the gain, that is, the relative importance, associated with each feature in each 1 of the 10 models trained. For each feature, we take the average gain across models as a final feature importance score. Note that we have both patient and drug features; thus, we have two vectors of feature importance scores, **i**_patients_ and **i**_drugs_. In a second step, we use the central matrices from the NMTF decompositions in our objective to link each feature to both genes and pathways. Specifically, we compute the projection of entity *x* in either drug or patient space with $G_{x}^{p}=G_{x}S_{t}$, where *S_t_* is either $S_{dt}$, for the drug space, or $S_{exp}+S_{mut}$, for the patient space. We can then rank the importance of genes, or pathways, by taking the product of the projected embeddings with the feature importance vectors. Interestingly, the two rankings of genes that we obtained retrieve driver genes in IntOGen. We consider all driver genes regardless of cancer type and compute the AUROC and AUPRC scores of the two rankings. We obtain AUROC 0.72 and 0.63 (*p*-values $<10^{-20}$) and AUPRC 0.08 and 0.04 in drug and patient space, respectively, which indicate significant correlations between the set of driver genes and the rankings. We take a closer look at the highest ranked genes and pathways (Table 2).

**Table 2.** Top 5 genes and biological pathways associated to drug response prediction-based feature importance in both drug and patient spaces

	Drug space	Patient space
Genes	HSP90AA1	KRT19
	PIK3CA	KRT8
	EGFR	KRT18
	PTEN	CLDN4
	PRKACA	AGR2
Pathways	Signal transduction	Extracellular matrix organization
	Signaling by GPCR	Transport of small molecules
	GPCR downstream signaling	Degradation of the extracellular matrix
	Metabolism	Signal transduction
	G alpha (i) signaling events	Response to elevated platelet cytosolic Ca^2+^

Interestingly, eight of the genes identified in Table 2 have been linked to cancer response to general, or specific treatments (HSP90AA1,^49^ PIK3CA,^50^ EGFR,^51^ PTEN,^52^ PRKACA,^53^ KRT19,^54^ CLDN4,^55^ and AGR2^56^), and the remaining two have been associated to cancer prognosis (KRT8^57^ and KRT18^58^). Put together, the results indicate that our model assigns meaningful importance to features. This is further corroborated when investigating the predicted pathways. We observe that most pathways are linked to external signaling, notably G protein-coupled receptors signaling. Signal transduction is naturally critical to drug response, as it is the route through which drugs interact with a cell.^59,60^ The state of the extracellular matrix also plays an important role, as it can prevent the penetration of small molecules into the cell, thus impairing pharmacologic treatments.^61^ Thus, our model learns to weigh meaningful features that relate to biological processes involved in drug mechanisms of actions.

## Conclusion

We introduce a two-step framework to perform data integration, feature reduction, and classification to uncover cancer-related knowledge. First, we develop an integrative NMF model to jointly embed entities in multiple connected latent spaces based on heterogeneous, diverse relational data between those entities. Note, that due to the wide range of data incorporated in our framework and the different levels of noise present in each, balancing strategies of the diverse objective functions should be investigated to improve the results further. Our model can easily be modified to accommodate such an approach.

We show that relative positions of entities in our latent spaces are consistent with what we know about them. For instance, we show that genes group in functional domains and are close to associated higher-order molecular structures (pathways and complexes) embedded in the same latent space. Patients tend to be closer to other patients having the same diagnosis and to the diagnosis itself. By taking a pan-cancer approach, we are able to identify groups of patients with similar molecular manifestations spanning various cancers, confirming that cancer classification may need to be rethought on a global scale and the need for initiatives such as PCAWG.^7^

Based on known drug indications for the treatment of each cancer type and known cancer type driver genes, we train decision trees through which we can predict relevant new associations for each cancer type. Due to the joint embedding of different entities in the same latent space, we hypothesized that decision trees trained to identify associations with one type of entities could be repurposed to derive associations with other, less-studied, entities. In this way, we can uncover biological mechanisms affected by each cancer type, such as genes whose expressions are significantly correlated with patient's survival (Supplementary Fig. S5). We also identify pathways that can be linked to cancer in the literature. Furthermore, our results suggest that cellular response to stress (pathway R-HSA-2262752) plays an important role in breast cancer. Similarly, we find that *FGFR* signaling mediated by *SHC* (pathways R-HSA-5654688, R-HSA-5654699, and R-HSA-5654719) is implicated with multiple cancer types; the precise role of SHC in these pathways has not been elucidated yet.

Interestingly, our work opens the door for actionable precision medicine. Through the joint embedding of cancers and patients, decision trees trained on high-level knowledge about cancer types can be repurposed to help identify patient-specific information, such as potential drug treatment. Furthermore, our model is able to capture the underlying information relevant to the characterization of patients' response to drug treatment. However, as the biological validation of such predictions is difficult, requiring cell line experiments or clinical trials, we leave it for future work.

Our framework is general and flexible and can accommodate additional and different data. New families of entities can be naturally added to the framework and could be illustrated by new nodes in Figure 1a. For instance, microbiomics, which is known to impact response to drugs,^62^ can be easily included in the general framework by adding a new node representing microbes in Figure 1a. Additional data connections between entities already in the framework, such as gene methylation data or gene copy number variation data, would add subobjectives to the optimization problem (represented by edges in Fig. 1a). While we focus on cancer in this study, our work paves the way for general cross-disease analysis, which could be useful to identify treatment repurposing based on molecular similarities among medical conditions. Alternatively, tasks such as drug side effects or drug combination synergy prediction can be addressed using the embedding framework as a basis with a specific machine learning model (e.g., boosted decision trees), taking the embedding as inputs.

## Data Availability

The data are available at https://life.bsc.es/iconbi/context_aware_embeddings/index.html

## Code Availability

The c++ library for the joint factorization is available at github.com/tgaudelet/nmfif

The analysis code, including baselines methods, is available at https://life.bsc.es/iconbi/context_aware_embeddings/index.html

## Supplementary Material

Supplemental data

## Abbreviations Used

ARI: adjusted rand index
AUPRC: area under the precision recall curve
AUROC: area under the receiver operating characteristic
BLCA: bladder urothelial carcinoma
BNNR: bounded nuclear norm regularization
BRCA: breast invasive carcinoma
CCGD: Candidate Cancer Gene Database
CESC: cervical squamous cell carcinoma
COAD: colon adenocarcinoma
CTD: comparative toxigenomics database
DRRS: Drug Repositioning Recommendation System
GBM: glioblastoma multiforme
GO-BP: Gene Ontology-Biological Processes
GO-CC: Gene Ontology-Cellular Component
GO-MF: Gene Ontology-Molecular Function
HNSC: head and neck squamous cell carcinoma
ICGC: International Cancer Genome Consortium
KIRC: kidney renal clear cell carcinoma
KIRP: kidney renal papillary cell carcinoma
LAML: acute myeloid leukemia
LGG: low grade glioma
LUSC: lung squamous cell carcinoma
MBiRW: measure-based bidirectional random walks
NBI: network based integration
NMF: non-negative matrix factorization
NMFR: non-negative matrix factorization reconstruction
NMTF: non-negative matrix tri-factorization
PCAWG: Pan-Cancer Analysis of Whole Genome
PPI: protein–protein interaction
READ: recum adenocarcinoma
SKCM: skin cutaneous melanoma
SNMTF: symmetric non-negative matrix tri-factorization
SNV: single-nucleotide variants
STAD: stomach adenocarcinoma
TCGA: The Cancer Genome Atlas
t-SNE: T-distributed stochastic neighbor embedding
UCEC: uterine corpus endometrial carcinoma
UMAP: Uniform Manifold Approximation and Projection
