# Supplementary material for: Integrative Data Analytic Framework to Enhance Cancer Precision Medicine
Source: Netw Syst Med. 2021 Mar 18;4(1):60–73. doi: 10.1089/nsm.2020.0015 (PMC8006589; doi:10.1089/nsm.2020.0015)
Supplement: Supplemental data [file Supp_Data.docx]

Supplement: Context-Aware Embedding Framework for Pan-Cancer Analysis and Precision Medicine

Thomas Gaudelet^1^, Noël Malod-Dognin^1,2^, and Nataša Pržulj^1,2,3,*^

^1^Department of Computer Science, University College London, London, WC1E 6BT

^2^Barcelona Supercomputing Center (BSC), Barcelona, 08034 Spain

^3^ICREA, Pg. Lluís Companys 23, 08010 Barcelona, Spain

## 1 Supplementary Methods

### 1.1 Data source and processing

We downloaded protein-protein interactions (PPI) data from Biogrid (version 3.5.176). We only keep interactions that have been validated experimentally using yeast-to-hybrid or affinity capture techniques. We obtained protein complexes data from CORUM and Reactome databases (both accessed in April 2019). Reactome is also used to collect all existing pathways of which we only keep pathways that have a traceable author statement (TAS). We further remove disease pathways which are only relevant in the associated disease context.

Patients data are obtained from the DCC Data release of the International Cancer Genome Consortium. We collected patients from 21 cancer cohorts from TCGA studies (see Table 1) and kept patients that have RNA-sequencing data, which adds up to 7,998 patients. We consider all Single Nucleotide Variations (SNV) reported in the data releases.

We consider the set of 15,224 genes whose transcripts are measured for by the RNA-sequencing technology across all datasets and that have at least one PPI with another selected gene according to BioGrid data. We derive a gene expression vector from RNA-seq measurement for each patient that is normalised to Transcripts Per Million (TPM) and rescaled using logarithm in base 2, i.e. the expression score of a gene is given by $\log_{2} (TPM+1)$. Note that we did not correct the data for potential confounders, but we verify a posteriori. To this end, we cluster the full cohort, as well as each cancer type cohort, into two clusters based on embeddings and measure: 1) bias toward age with a Mann-Whitney U statistical test between the two clusters, and 2) bias toward sex by using the ARI metric. We observe no significant bias towards these variables (see Supplementary Table 4). Note that 559 patients have do not have any mutation on any of the 15,224 genes considered.

Drug–target and chemical data is obtained from DrugBank (version 5.1.3) [35]. We consider all drugs that are approved, experimental, or investigational. Drugs chemical similarity is computed using the Tanimoto similarity [28] between drugs smile representations. While it does not assure similar functional impact, the Tanimoto coefficient is the default standard and has been used in virtual screenings to assess likelihood of a drug binding to a known target [5]. The details of the data used can be found in Table 2.

Genes’ annotations used for the enrichment analysis are obtained from Gene Ontology (GO)[4] (release 10/06/2019). We keep annotations that have an experimental evidence code (one of EXP, IDA, IPI, IMP, IGI, and IEP). We consider all three GO annotation subtypes separately: Biological Processes (GO–BP), Molecular Function (GO–MF) and Cellular Component (GO–CC). For each, we build a directed acyclic graph (DAG) that connects annotations based on “is a” relationships (we use the go-basic.obo file giving annotations relationships available on GO’s website). Then, we propagate the annotations for each gene up the corresponding DAG, which means that we add to the set of annotations of a gene the union of ancestors of the annotations. We remove annotations that annotate less than $0.1\%$, or more than $10\%$ of the 15,224 genes considered, i.e. we prune annotations that are either too rare, or too common. We give the statistics of annotations in Table 3

### 1.2 Non-negative Matrix Factorizations

Matrix factorizations approaches aim to approximate a matrix $X$ by the product of $n$ smaller matrices $F_{i},i\in\{1..n\}$, called factors, i.e. $X\approx\prod_{i} F_{i}$. Mathematically, this amounts to finding factors $F_{i}$, under user defined dimensional contraints, that minimize the equation $\parallel X-\prod_{i} F_{i}\parallel_{F}^{2}$, where represents the Frobenius norm of a matrix. Non-negative matrix factorizations techniques add a positivity contraint on the factors, i.e. $\forall i, F_{i}\geq0$.

The objective is to obtain lower dimensional representation that captures the essence of the data and can be used to identify missing entries through the matrix completion property. In this work, we use three variants of non-negative matrix factorisations approaches.

**NMF** decomposes a rectangular matrix in the product of two positive factors and , with $k\leq\min(m,n)$, such that $\parallel X-FG^{T}\parallel_{F}^{2}$ is minimized. With NMF, the embeddings, given by $F$ and $G$, of the two groups of entities whose relational data is given by $X$, are in the same latent space.

**NMTF** decomposes a rectangular matrix in the product of three positive factors , and , with $k_{1}, k_{2}\leq\min(m,n)$, such that $\parallel X-FSG^{T}\parallel_{F}^{2}$ is minimized.

**SNMTF** decomposes a symmetric matrix in the product of two positive factors and , with $k\leq n$, such that $\parallel X-GSG^{T}\parallel_{F}^{2}$ is minimized.

### 1.3 Our Integrative Framework

We propose a framework that jointly integrates all data sources together by using a mixture of non-negative matrix factorisations to obtain for each entity an embedding that takes into account the full context of our task. We minimize the following general objective function over all $G.$ and $S.$ factors:

(1)

$$+\parallel X_{ppi}-G_{g}S_{ppi}G_{g}^{T}\parallel_{F}^{2}+\parallel X_{pct}-G_{ct}G_{p}^{T}\parallel_{F}^{2}$$

$$+\parallel X_{ts}-G_{d}S_{ts}G_{d}^{T}\parallel_{F}^{2}+\parallel X_{dt}-G_{d}S_{dt}G_{g}^{T}\parallel_{F}^{2},$$

where, henceforth, each $X_{D}$ represents the matrix associated to data type $D$, see nomenclature in Table 2, each $G_{E}$ factor gives the embeddings of the entities of type $E$, with subscripts $g$, $p$, $ct$, $d$, $bp$, and $pc$ corresponding, respectively, to genes, patients, cancer types, drugs, pathways, and complexes. *S*. factors are optimized over, but not used for the analysis.

The integration of the various data sources is achieved by sharing factors across the NMF sub-objectives that constitute our global objective function . For instance, the factor $G_{g}$, corresponding to the genes embeddings, is shared by all decompositions that involve genes which corresponds to the factorisation of PPI data ($X_{ppi}$), the factorisations of patients molecular data ($X_{exp}$ and $X_{snv}$), the factorisation of drug–target data ($X_{dt}$), and the factorisations of higher-order biological entities ($X_{pc}$ and $X_{bp}$). Through this factor sharing and joint optimisation, the framework is able to harness the relevant information contained across the data sources to derive meaningful embeddings.

### 1.4 Optimization

The minimisation of the objective function given in Equation 1 is achieved through an iterative optimisation process. We use in our framework multiplicative update rules [21] designed to maintain non-negativity of all the factors in the decomposition.

We use an initialization strategy based on the truncated singular value decomposition (SVD) for all factors that has shown better performances than random initialization [8, 25] and has the advantage of giving deterministic solutions. Specifically, consider a factor involved in the decomposition of $l$ data matrices $X_{i}, i\in\{1..l\}$. Without loss of generality, we assume that $G$ is the right hand side factor in the decompositions, i.e. $\forall i, X_{i}\approx GF_{i}$. We denote by $U_{i}$ the right hand side term in the SVD decomposition of $X_{i}$ ($X_{i}=U_{i}S_{i}V_{i}^{T}$). We introduce $U_{i}^{+} = \max(U_{i},0)$ and $U_{i}^{-} = \max(-U_{i},0)$. We then denote by the matrix where each column is defined by

$$\tilde{U}_{i}(j) = \left\{\begin{matrix} \sqrt{s_{i}(j)}U_{i}^{+}(j), & \text{if}\parallel U_{i}^{+}(j)\parallel\geq\parallel U_{i}^{-}(j)\parallel\\ \sqrt{s_{i}(j)}U_{i}^{-}(j), & \text{otherwise} \end{matrix}\right.,$$

where $M(j)$ denotes the $j^{th}$ column of the matrix $M$ and $s_{i}(j)$ is the $j^{th}$ largest singular value of $X_{i}$. Factor *G* is then initalized as $\frac{1}{l}\sum_{i=1}^{l} \tilde{U}_{i}+\varepsilon$. We initialize the central matrix in NMTF decompositions to $I+\varepsilon$, where *I* denotes the identity matrix. Under the multiplicative update rules, any entries initialized to zero would stay null. Hence, we add a small $\varepsilon$ everywhere to allow all entries to vary.

The iterative optimisation is ran either for 200 epochs or until the relative variation of the objective function between two consecutive epochs is lower than $10^{-4}$, i.e. when where corresponds to the value of the objective function at iteration *t*.

### 1.5 Projecting new, unseen patients in our framework latent space

can be achieved by solving an objective function derived from the framework. Specifically, to find embeddings in our latent space for patients that were not seen by the model during the optimization process, we minimize the objective function

where $X_{x},x\in\{exp,snv\}$ represent the molecular data of the patients, $X_{pct}$ gives the patient diagnosis. The star superscripts ·* denote factors that are fixed in the original framework decomposition. Once this objective is minimized, *G_p_* gives the embeddings of the new patients in the latent space. We measure the quality of the embeddings of the new patients by quantifying if the patient is embedded close to its diagnosis and close to other patients having the same cancer in the original dataset. The first aspect is quantified with macro-F1 scores of a classifier that associate to each patient the closest cancer in the latent space. The second aspect is quantified with macro-F1 scores of a classifier that associates to each patient the diagnosis that is most represented among the 10 nearest patients in the latent space.

### 1.6 Boosted decision tree

In the main document, we used boosted decision trees to predict cancer type associations with entities that are part of our framework based on the embeddings derived from the Joint NMF optimisation step. A decision tree classifier partitions the input data iteratively based on features. Boosting signifies deriving a strong classifier from the serial associations of weak classifiers. In our case, the base classifiers are decision trees. The boosted algorithm iteratively adds decision trees to the classifier with the aim of reducing the error of the previous classifier[29].

We discuss here the implementation details that we used. First, we use boosted decision trees from the xgboost package [9]. Boosted decision trees have different hyperparameters that control various aspect of the algorithm: $\eta$ controls the learning rate, $\gamma$ corresponds to a threshold under which a leaf node of the decision tree is not split anymore, the maximal depth of a decision tree, and $\lambda$ controls the L2-regularization (for more details see [9]). We perform a $10$-fold cross-validation to fix those hyperparameters with $\eta\in\{0.25,0.5,0.75\}$, $\gamma\in\{0,10\}$, max depth$\in\{6,12\}$, and $\lambda\in\{1,10,100\}$. The best set of parameters is chosen as the one that leads to the classifier with the highest AUROC score in the associated task. Note that we also use early-stopping during training with an $80\%$/$10\%$/$10\%$ train/validation/test split of our data. We use all $10$ classifiers trained during the cross-validation process to derive an association score for each possible pair. To ensure that the scoring of the $10$ classifiers is comparable, we rescale the output scores to have 0 mean and unit variance. The average of all classifier scores then gives the final association score of an entity pair.

### 1.7 Baselines

We contrast the performances of our trained boosted decision tree with those of the state-of-the-art methods for the prediction of cancer type associations with genes and drugs. We chose baselines based on the availability of source code (or detailed implementation description), the quality of reported performances, and the concordance of input data with ours. Our implementation of each method is available in the Supplementary Files. When a method requires hyperparameters tuning, the criterion used to identify the best set of hyperparameters is always the AUROC score of the classifier in the associated task.

**Non-negative Matrix Factorization Reconstruction (NMFR)** is based on the reconstruction of the data after factorizations and is the simplest approach based on our framework. The idea is based on the matrix completion property observed in matrix factorizations methods[27]. Here, we propose a simple method that makes use of the link between factors to extract entities’ association scores. For instance, cancer–gene association scores, $CG$, are given by

$$CG=G_{ct}G_{p}^{T}G_{p}\frac{\left(S_{exp}+S_{snv}\right)}{2}G_{g}^{T},$$

where entry $(i, j)$ of matrix $CG$ gives the association score between cancer type $i$ and gene $j$. Cancer–drug association scores, $CD$, are given by

$$CD=G_{ct}G_{p}^{T}G_{p}\frac{\left(S_{exp}+S_{snv}\right)}{2}G_{g}^{T}G_{g}S_{dt}G_{d}^{T},$$

where entry $(i,j)$ of matrix $CD$ gives the association score between cancer type $i$ and drug $j$.

Performances are measured by how well those association scores correlate to IntOGen cancer–driver data and DrugCentral cancer–drug data using AUROC and AUPRC.

**MBiRW** [24] was proposed to identify potential new indications for the existing drugs. The method is based on a bi-directional random walk using a drug similarity network, a disease similarity network, and a bipartite network connecting diseases to drugs. The authors report good performances against known ground-truth relative to the competing methods and manually validate de novo predictions.

Here, the drug similarity network adjacency matrix is given by the drug Tanimoto similarities matrix $X_{ts}$. We define the cancer similarity network $X_{cc}$ based on a molecular similarity between cancers. We first associate to each cancer two molecular signatures given by the average of patients gene expression data and SNV data. From each type of data, we define a cancer similarity network that corresponds to the cosine similarity of their molecular signatures. We denote by $X_{cc}$ the final cancer similarity network corresponding to the average of those two similarity networks. Note that the authors use a different disease similarity matrix, the source of which is currently offline. Finally, we use cancer–drug data from DrugCentral to define a bipartite network in which an entry is set to 1 to indicate an association between the corresponding drug and cancer, and 0 otherwise.

The authors propose an iterative method that follows the step given in Algorithm 1.

We perform a 10-fold cross-validation to select hyperparameters $\alpha\in\{0.1,0.3,0.5,0.7,0.9\}$ and $M \in\{2,5,10,20\}$ (note that the authors set $M=2$, and search for $\alpha$). In each run, $10\%$ of known cancer–drug associations are masked in the input to the algorithm and we evaluate how well MBiRW is able to retrieve those.

**DRRS** [23] was proposed to identify potential new indications for the existing drugs as well. The method is based on the matrix completion property of Singular Value Thresholding Algorithm (SVT) using a drug similarity matrix, a disease similarity matrix, and a disease–drug indication matrix. The authors report good performances against known ground-truth relative to the competing methods and further use their methods to predict indications for new drugs, validating novel associations.

**Data:** cancer–cancer network $X_{cc}$, drug–drug network $X_{ts}$, cancer–drug network $CD$, parameter *α*, maximum number of iterations *M*

**Result:** cancer–drug associations scores $O$

$DD=D_{ts}^{-\frac{1}{2}}X_{ts}D_{ts}^{-\frac{1}{2}};$where $D_{ts}$ is a diagonal matrix where entry $D_{ts}(i,i)=\sum_{j} X_{ts}(i,j)$

$CC=D_{cc}^{-\frac{1}{2}}X_{cc}D_{cc}^{-\frac{1}{2}};$where $D_{cc}$ is a diagonal matrix where entry $D_{cc}(i,i)=\sum_{j} X_{cc}(i,j)$

$R_{0}=\frac{CD}{\sum CD}$; where $\sum CD$ gives the number of non-zero entries in $CD$

$O=R_{0}$; $n_{i}ter=0$;

**while** $n_{iter}\leq M$ **do**

$L=\alpha\cdot O\cdot DD+(1-\alpha)\cdot R_{0}$;

$R=\alpha\cdot CC\cdot O+(1-\alpha)\cdot R_{0}$;

$O=\frac{L+R}{2}$;

$$n_{iter}=n_{iter}+1;$$

**end**

**Algorithm 1:** MBiRW algorithm.

For our purposes, we use the Tanimoto drug similarity matrix $X_{ts}$, the cancer–cancer similarity matrix derived from ICGC molecular data $X_{cc}$, and cancer–drug associations from DrugCentral $X_{cd}$. The authors define the block matrix $A$

$$A=\left(\begin{matrix} X_{ts} & X_{cd}^{T} \\ X_{cd} & X_{cc} \end{matrix}\right),$$

and feed it to the SVT algorithm. The maximum number of epochs is set to the minimum between the number of cancers and the number of drugs. The iteration with the highest AUROC gives the final predictions. We perform a $10$-fold cross-validation to evaluate the performance of the algorithm. In each iteration, we mask $10\%$ of known cancer–drug associations and evaluate how well the algorithm retrieves them.

**BNNR** [38] was developed for re-purposing of drugs. The method is also based on the completion property of SVT. The algorithm follows the steps given in Algorithm 2. Compared to DRRS, BNNR incorporates a regularisation term to balance the approximation error and the rank properties and thus can handle the noisy drug–drug and disease–disease similarities. It also adds a constraint that clips the association scores to the interval $[0,1]$. The authors found that those additions benefited performances compared to DRRS for retrieval of known associations. They further manually validated the top-scoring associations through literature curation.

We perform a $10$-fold cross-validation to evaluate the performance of the algorithm and fix the hyperparameters $\alpha\in\{0.1,1,10,100\}$ and $\beta\in\{0.1,1,10,100\}$.

**Network Based Integration (NBI)** [30] was developed to identify cancer-related genes that are not necessarily mutated or differentially expressed. The method is based on network heat diffusion process over a molecular network. The original paper focuses on a specific cancer for which they collect differential gene expression data and SNV data. The authors assess performances by first measuring how accurately their method retrieves known cancer driver genes and then validate novel cancer–gene associations.

Network heat diffusion is defined by the iterative update of scores $X^{0}$ associated to the network’s nodes following the equation

$$X^{n+1}=\alpha WX^{n}+(1-\alpha)X^{0},$$

where *W* denote the network data and $X^{n}$ corresponds to the updated scores after *n* iterations. The iterative process terminates when $\parallel X^{n+1}-X^{n}\parallel_{2} < 10^{-6}$.

**Data:** block matrix $A$, hyperparameters $\alpha$ and $\beta$

**Result:** cancer–drug associations scores $W$

$tol_{1}=2\cdot1e^{-3}$; $tol_{2}=1e^{-5}$;

$M=300$; $n_{iter}=0$;

$s_{1}=1$; $s_{2}=1$;

$X=A$; $Y=X$; $W=X$;

$mask=T>0$;

**while** $n_{iter}\leq M$ *or* $tol_{1}<s_{1}$ *or* $tol_{2}<s_{2}$ **do**

$F=\frac{1}{b}(Y+\alpha T)+X$;

$W=F-\frac{\alpha}{\alpha+\beta}F\odot mask$;

$$W[W<0]=0;$$

$$W[W>1]=1;$$

$$X_{t}=svt(W-\frac{1}{\beta}Y,\frac{1}{\beta});$$

$$Y=Y+\beta(X_{t}-W);$$

$$s_{t}=s_{1};$$

$$s_{1}=\frac{\parallel X_{t}-X\parallel_{F}}{\parallel X\parallel_{F}};$$

$$s_{2}=\frac{|s_{1}-s_{t}|}{\max(1,|s_{t}|)};$$

$$X=X_{t};$$

$$n_{iter}=n_{iter}+1;$$

**end**

**Algorithm 2:** BNNR algorithm.

The authors set $W$ to the PPI normalized adjacency $W=\Delta^{-\frac{1}{2}}X_{ppi}\Delta^{-\frac{1}{2}}$, where $\Delta^{-\frac{1}{2}}$ is a diagonal matrix where entry $i$ corresponds to the degree of gene $i$ in the PPI network. The authors use the diffusion process both on patients differential gene expression and SNVs, obtaining two diffused vector scores per patient. They then handcraft $13$ cancer-specific features for each gene based on the results. Those features are then used as input to a logistic regression classifier trained to predict known cancer drivers.

As differential gene expression is not available to us, we use the same gene expression values that are input to our framework. Since our analysis is across cancers, we compute gene features for each cancer types, i.e. each cancer–gene pair is associated with a $13$-dimensional feature vector. The method has two hyperparameters: *α* for the heat diffusion process, and *C* that controls regularisation of the logistic classifier. We perform a $10$-fold cross-validation procedure to pick the best pair of hyperparameters, with $\alpha\in\{0.25,0.5,0.75\}$ and $C \in\{0.01,1,100\}$, and to evaluate the performance of a logistic regression classifier trained on those features to predict cancer-specific driver genes.

**LOTUS** [11] is a method that achieved the state-of-the-art results for the more specific tasks of identifying oncogenes and tumour-suppressing genes. Each task is tackled separately, and each with $3$ different gene features that are not available to us. However, the method can be adapted to the simpler task of retrieving cancer driver genes. To this end, we use gene expression data, SNVs, and gene methylation data, that are available in ICGC, as gene features. A patient’s gene methylation is defined as the average beta value of all associated CpG islands.

The authors of LOTUS propose both a cancer-specific framework and a pan-cancer framework; we use the latter here. The method revolves around the Support Vector Machine (SVM) algorithm. The authors first define for each sample–gene pair $3$ features that are then averaged across all samples to give the final gene features. In their work, the final features correspond to the number of damaging missense mutations, the total number of missense mutations, and the entropy of the spatial distribution of the missense mutations on each gene, for the prediction of oncogenes. For the prediction of tumour-suppressing genes, the features are the number of frameshift mutations, the number of loss-of-function mutations, and the number of splice site mutations. Note that when defining those features, the authors do not differentiate across cancer types. In our case, the features correspond to the mutation frequency, the average gene expression, and the average gene methylation across all samples. To ensure that those features are comparable, we normalise the distributions to have 0 mean and unit variance.

The authors then define both a gene kernel $K_{g}$ and a cancer kernel $K_{c}$. The gene kernel is defined as the average of a kernel corresponding to a gene similarity matrix derived from $3$-dimensional features defined above and a kernel derived from the PPI network. We have

$$K_{g}=\frac{1}{2}\left(\Phi\Phi^{T}+e^{-L}\right),$$

where represents the gene features and $L$ is the normalized Laplacian of the PPI network, $L=I-D^{-\frac{1}{2}}X_{ppi}D^{-\frac{1}{2}}$, $I$ represents the identity matrix and $D$ the diagonal matrix with entries corresponding to the degree of each node in the PPI network. The cancer kernel is defined as the sum of three kernels

$$K_{c}=\frac{1}{3}\left(I+J+X_{cc}\right),$$

where $I$ represents the identity matrix, $J$ corresponds to the matrix filled with ones, and $X_{cc}$ is a cancer similarity matrix. As above, we use the cancer similarity matrix defined based on cancers molecular similarities.

The final kernel $K$ for (cancer,gene) pairs used for pan-cancer analysis is defined by

$$K\left((c,g),(c',g')\right)=K_{c}(c,c')\times K_{g}(g,g'),$$

where *c* and $c'$ represent cancers and *g* and $g'$ represent genes. The hyperparameter of the model corresponds to the regularisation coefficient $C$ of the SVM algorithm. Due to the large size of the full kernel $K$, we use the same strategy as the authors of LOTUS and randomly sample negative (cancer,gene) pairs from all (cancer,gene) pairs that are not reported in IntOGen. This effectively boils down to using a submatrix of kernel *K* as input that contains as many positive (cancer–driver associations) and negative pairs. As before, we perform cross-validation to pick $C \in\{1,10,100,1000\}$ and evaluate the performance of the method on our task.

**Subdyquency** [33] is a method based on random walks on a network to identify cancer drivers. It achieves the state-of-the-art results for the retrieval of known cancer drivers. The framework is defined for specific cancers and we extend it to pan-cancer. In their framework, the authors build a network between “outlier” and mutated genes. The outlier genes are genes whose expression is significantly different with respect to the cohort. They correspond to genes with absolute z-score strictly greater than 2. The set of outlier (mutated) genes is defined as all genes being at least outlier (mutated) for one patient. Here, we consider all cancers together to define those sets. Directed interactions between genes are obtained from the Functional Interactions (FI) network [36] derived from Reactome [15]. We downloaded the 2019 version of the FI network. The authors define a bipartite graph between the two sets of genes whose edges correspond to directed links in the FI network. The edge weights are defined based on the localisation of proteins in a cell as given by the COMPARTMENT database [7] (see the original paper for details). As done by the authors, we downloaded all data relating to human regardless of evidence type (obtained in April 2020). We denote the adjacency matrix of this bipartite graph with , where $n_{m}$ and $n_{o}$ represent the number of mutated and outlier genes, respectively. Then, for each patient $p$, the authors define a feature vector for the outlier gene set, denoted by $O_{p}$, and another one for the mutated gene set, denoted by $M_{p}$. Specifically, consider gene $i$ in the mutated set. If gene $i$ is mutated for patient $p$, then $M_{p}(i)$ is set to the mutation frequency of gene $i$ in the cohort of patients having the same cancer as $p$, and $0$ otherwise. For gene $j$ in the outlier set, if $j$ is not an outlier for patient $p$, then $O_{p}(j)$ is set to $0$, if $j$ is an outlier and is also in the mutated set, then $O_{p}(j)$ is set to $M_{p}(j)$, else it is set to the outlier frequency of $j$ across the set of patients having the same cancer as patient $p$. The authors then propose the three steps procedure simulating a random walk on the bipartite graph using both feature vectors

$$R_{p}^{m}=\alpha M_{p}+(1-\alpha)WO_{p},$$

$$R_{p}^{o}=\alpha O_{p}+(1-\alpha)W^{T}R_{p}^{m},$$

$$R_{p}^{m}=\alpha M_{p}+(1-\alpha)WR_{p}^{o},$$

where $\alpha\in[0,1]$ is the sole hyperparameter of the model. The final cancer–gene scores are derived by summing the $R_{p}^{m}$ vectors across patients. Higher scores indicate a stronger associations between a cancer and a gene. We perform a cross-validation to pick $\alpha\in[0,1]$ and evaluate the performance of the method on our task.

## 2 Supplementary Results

### 2.1 Cancer–drug associations

Due to space limitations, we discuss here the supporting literature for the remaining predicted drugs in Figure 3.b. of the main article.

DB12202 (Zalutumumab) targets EGFR gene and is investigated for the treatment of Squamous Cell Cancer and Head and Neck Cancer.

DB05374 (Rindopepimut) is a drug investigated for the treatment of brain cancers. It targets the mutant protein EGFRv3, which has recently been identified as a target in lung cancer therapy as well as [41].

DB01269 (Panitumumab) is approved for the treatment of EGFR-expressing colorectal carcinoma. Since EGFR is often also involved in lung cancers, the predicted associations here are relevant.

DB05931 (Pegdinetanib) is an investigational drug for the treatment of unspecified cancers. It binds to gene VEGFR-2 regulating primary tumour angiogenesis pathways, thus blocking ligands from binding to VEGFR-2.

DB06186 (Ipilimumab) is an approved drug for the treatment of multiple cancers, such as renal cell carcinoma, melanoma, and colorectal cancer. It binds CTLA4 to block the T-cell inhibition signal pathway. Erfani *et al.* [14] suggested that therapies targeting CTLA4 might be beneficial to lung cancer patients.

DB00011 and DB00018 (interferon alpha-n1 and interferon alpha-n3) are proteins that both targets interferon alpha/beta receptors 1 and 2. A similar protein, interferon alpha-2b, is among the approved treatments of SKCM. The mechanisms of action of these protein-based treatments are identical according to Drugbank. This supports the prediction of both DB00011 and DB00018 for the treatment of SKCM.

### 2.2 Cancer–gene associations

Due to space limitations, we discuss here the supporting literature for the remaining predicted genes in Figure 3.d. of the main article.

We predict that HERC1 is associated with BRCA, COAD, and READ. The associations with COAD and READ are already reported in CCGD. Furthermore, HERC1 has been linked to migration and invasion of breast cancer cells [16]. We further observe, with a logrank statistical test ($0.05$ cut-off), that higher than average expression of HERC1 in our BRCA cohort leads to significantly lower survival rates (pvalue $0.0498$; see Supplementary Figure 2.d). Inversely, lower than average expression of HERC1 in our READ cohort indicates significantly lower survival rates (p-value $0.008$; see Supplementary Figure 2.e).

Both NCOA3 and CHD6 are linked to BRCA in CCGD. We further observe here, with a logrank statistical test, that higher than average expression of those genes indicates lower survival rates in our BRCA cohort with p-values $0.0014$ and $0.0155$, respectively (see Supplementary Figure 2.f/g).

We predict that SIN3A gene is linked to BRCA, which is supported by existing literature [12, 22].

SRC is a proto-oncogene linked to colon cancer, according to NCBI. It has notably been connected to breast cancer in the scientific literature [13].

PPARG has been identified as a potential target for cancer treatment and prevention [3]. It has also been associated with the induction of apoptosis in breast cancer cells [39]. This is consistent with our logrank statistical analysis which shows that higher than average expression of PPARG in our BRCA cohort is associated with significantly higher survival rates (p-value $0.016$; see Supplementary Figure 2.g).

### 2.3 Cancer–complex associations

Due to space limitations, we discuss here the predicted protein complexes.

To obtain the association score between a cancer type and a protein complex, we simply feed the concatenation of the normalised embeddings of both entities to the $10$ decision trees trained to predict cancer–gene associations. The average of the standardised scores across all decision trees gives the final association score.

To the best of our knowledge, there are no comprehensive database reporting associations between cancers and protein complexes. Thus, we are unable to provide global validation scores for our predictions. We proceed by validating the top $10$ scoring protein complexes manually (see Table 6) below.

IL6:sIL6R:IL6RB:JAKs complex plays a role in interleukin 6 signalling, which is linked to cancer [19]. The complex is associated with JAK family of kinases; themselves tied to cancer [34]. p-7Y-RUNX1:PTPN11 complex is involved in the regulation of RUNX1 expression and activity. RUNX1 has been linked to various cancer, sometimes with opposite effects [1]. However, regardless of its precise role in a given cancer, the regulation of RUNX1 appear to be of critical importance as over- or under-expression can have an important impact on the development of cancer[18]. Tyrosine phosphorylated IL6 receptor hexamer:Activated JAKs:Tyrosine/serine phosphorylated STAT1/3 complex (R-HSA-1112759) is involved with interleukin 6 signalling and more specifically serine phosphorylation of STAT family of transcription factors. We have seen previously that interleukin 6 signalling has been linked to cancer. Furthermore, STAT has been linked to various cancers, including breast cancer (BRCA) that our results associate to the protein complex [10].

Integrin alpha2bbeta3:SRC complex plays a role in integrin signalling, more specifically in the phosphorylation of SRC kinase. Our prediction is supported by the fact that integrin alpha2bbeta3 is part of the oncogenic MAPK signalling pathways[15] which includes inactive SRC (phosphorylated Y530).

IL6:Tyrosine phosphorylated hexameric IL-6 receptor:Activated JAKs:p-Y546,Y584-PTPN11 complex (R-HSA-1112753) is implicated in MAPK1/MAPK3 signalling. Both MAPK1 and MAPK3 are identified as driver genes in some cancers[1] and phosphorylated PTPN11 (p-Y546,Y584-PTPN11) is linked to PI3K/AKT signalling in cancer[15]. Furthermore, the MAPK pathway, through interplay with PI3K, has been linked to breast cancer [17] and leukemia [37].

Tyrosine phosphorylated IL6ST:Activated JAKs complex (R-HSA-1112563) is linked to both interleukin 6 signalling, MAPK1/MAPK3 signalling, and phosphorylated PTPN11. Based on the discussion above, this complex is relevant to breast cancer.

SAM68:p120GAP complex has been linked to the insulin receptor signalling pathway. More specifically, Sánchez-Margalet *et al.* [32] reports that Sam68 is associated with p120GAP after insulin stimulation and links GAP to the PI3K pathway. We have already seen that PI3K pathway has be linked to breast cancer in previous work. It is also the case that insulin receptor signalling plays a role in breast cancer [6].

JAKs:OSMR complex is also involved in Interleukin-6 family signalling. Furthermore, it has been suggested that OSMR and JAK/STAT3 signalling can promote breast cancer progression [20].

IL6ST:JAKs complex is a subunit of IL6:sIL6R:IL6RB:JAKs that is involved in signalling by interleukins. The same supporting evidence links it to cancer.

Me260-ESR1:STRN:ESTG:MyrG-pY419 SRC:PI3K alpha complex (R-HSA-9632399) is part of the extra-nuclear estrogen signalling pathway which has been linked to tumour progression and metastasis [31]. Furthermore, protein MyrG-p-Y419-SRC, bound to PI3K in the complex, is associated with various cancer pathways[15], including PI3K/AKT signalling in cancer.

The literature curation highlights that our predicted associations between protein complexes and cancer types are supported by the existing literature. Thus, the analysis demonstrates the validity of our re-purposing approach, as well as the ability of our framework to extract protein complexes linked to cancer.

### 2.4 Cancer–pathway associations

Due to space limitations, we discuss here the supporting literature for the remaining biological pathways in Table 2 of the main article.

SHC-mediated cascade:FGFR1 pathway (R-HSA-5654688), SHC-mediated cascade:FGFR2 pathway (R-HSA-5654699), and SHC-mediated cascade:FGFR4 pathway (R-HSA-5654719) are all three sub-pathways of FGFR signalling pathway which has been connected to cancer [2]. The role of SHC in this is unclear, but observations suggest that it might contribute to activation of the MAPK pathway [15].

CD28 dependent PI3K/Akt signalling pathway (R-HSA-389357) is associated with cell growth and survival, roles that are critical in the development of cancer. The PI3K/Akt signalling pathway has been identified as a potential therapeutic target for cancers, including breast cancer [26]. Furthermore, mutation of CD28 receptors has recently been linked to increased risk in breast cancer [40].

Our results suggest that activated NTRK3 signals through PI3K pathway (R-HSA-9603381) is associated with breast cancer. The activation of NTRK3 correlates with activating phosphorylation of AKT, the principal mediator of PI3K signalling. Thus, this association is directly related to cancer, as shown in a previous discussion.

TFAP2 (AP-2) family regulates transcription of growth factors, and their receptors pathway (R-HSA-8866910) is associated with breast cancer through ESR1 and ERRB2. The entry for the pathway in Reactome [15] details the link between TFAP2 family and expression of ESR1 in breast cancer.

## 3 Supplementary figures and tables

**References**

[1] Kenneth L Abbott, Erik T Nyre, et al. The Candidate Cancer Gene Database: a database of cancer driver genes from forward genetic screens in mice.  *Nucleic Acids Research*, 43(Database issue):D844–8, jan 2015.

[2] Imran Ahmad, Tomoko Iwata, and Hing Y Leung. Mechanisms of fgfr-mediated carcinogenesis.  *Biochimica et Biophysica Acta (BBA)-Molecular Cell Research*, 1823(4):850–860, 2012.

[3] Clinton D Allred and Michael W Kilgore. Selective activation of ppar$\gamma$ in breast, colon, and lung cancer cell lines.  *Molecular and Cellular Endocrinology*, 235(1-2):21–29, 2005.

[4] Michael Ashburner, Catherine A Ball, et al. Gene ontology: tool for the unification of biology. *Nature Genetics*, 25(1):25–29, 2000.

[5] Dávid Bajusz, Anita Rácz, and Károly Héberger. Why is tanimoto index an appropriate choice for fingerprint-based similarity calculations? *Journal of cheminformatics*, 7(1):20, 2015.

[6] Antonino Belfiore and Francesco Frasca. Igf and insulin receptor signaling in breast cancer. *Journal of Mammary Gland Biology and Neoplasia*, 13(4):381–406, 2008.

[7] Janos X Binder, Sune Pletscher-Frankild, et al. Compartments: unification and visualization of protein subcellular localization evidence. *Database*, 2014, 2014.

[8] Christos Boutsidis and Efstratios Gallopoulos. Svd based initialization: A head start for nonnegative matrix factorization. *Pattern Recognition*, 41(4):1350–1362, 2008.

[9] Tianqi Chen and Carlos Guestrin. Xgboost: A scalable tree boosting system. In *Proceedings of the 22nd ACM SIGKDD International Conference on Knowledge Discovery and Data Mining*, pages 785–794, 2016.

[10] Charles V Clevenger. Roles and regulation of stat family transcription factors in human breast cancer. *The American Journal of Pathology*, 165(5):1449–1460, 2004.

[11] Olivier Collier, Véronique Stoven, and Jean-Philippe Vert. Lotus: A single-and multitask machine learning algorithm for the prediction of cancer driver genes. *PLoS Computational Biology*, 15(9):e1007381, 2019.

[12] Stephanie J Ellison-Zelski and Elaine T Alarid. Maximum growth and survival of estrogen receptor-alpha positive breast cancer cells requires the sin3a transcriptional repressor. *Molecular Cancer*, 9(1):263, 2010.

[13] Beatrix Elsberger. Translational evidence on the role of src kinase and activated src kinase in invasive breast cancer. *Critical Reviews in Oncology/Hematology*, 89(3):343–351, 2014.

[14] Nasrollah Erfani, Shayesteh Mofakhami Mehrabadi, et al. Increase of regulatory t cells in metastatic stage and ctla-4 over expression in lymphocytes of patients with non-small cell lung cancer (nsclc). *Lung Cancer*, 77(2):306–311, 2012.

[15] Antonio Fabregat, Steven Jupe, Lisa Matthews, Konstantinos Sidiropoulos, Marc Gillespie, Phani Garapati, Robin Haw, Bijay Jassal, Florian Korninger, Bruce May, Marija Milacic, Corina Duenas Roca, Karen Rothfels, Cristoffer Sevilla, Veronica Shamovsky, Solomon Shorser, Thawfeek Varusai, Guilherme Viteri, Joel Weiser, Guanming Wu, Lincoln Stein, Henning Hermjakob, and Peter D’Eustachio. The reactome pathway knowledgebase. *Nucleic Acids Research*, 46(D1):D649–D655, 2018.

[16] Natsuka Goto, Hiromi Hiyoshi, et al. Estrogen and antiestrogens alter breast cancer invasiveness by modulating the transforming growth factor-$\beta$ signaling pathway. *Cancer Science*, 102(8):1501–1508, 2011.

[17] Huizhong Hu, Alexey Goltsov, et al. Feedforward and feedback regulation of the mapk and pi3k oscillatory circuit in breast cancer. *Cellular Signalling*, 25(1):26–32, 2013.

[18] Kevin A Janes. Runx1 and its understudied role in breast cancer. *Cell Cycle*, 10(20):3461–3465, 2011.

[19] Neeraj Kumari, BS Dwarakanath, et al. Role of interleukin-6 in cancer progression and therapeutic resistance. *Tumor Biology*, 37(9):11553–11572, 2016.

[20] Lore Lapeire, An Hendrix, et al. Cancer-associated adipose tissue promotes breast cancer progression by paracrine oncostatin m and jak/stat3 signaling. *Cancer Research*, 74(23):6806–6819, 2014.

[21] Daniel D Lee and H Sebastian Seung. Algorithms for non-negative matrix factorization. In *Advances in Neural Information Processing Systems*, pages 556–562, 2001.

[22] Monica J Lewis, Jianzhong Liu, et al. Sin3a and sin3b differentially regulate breast cancer metastasis. *Oncotarget*, 7(48):78713, 2016.

[23] Huimin Luo, Min Li, et al. Computational drug repositioning using low-rank matrix approximation and randomized algorithms. *Bioinformatics*, 34(11):1904–1912, 2018.

[24] Huimin Luo, Jianxin Wang, et al. Drug repositioning based on comprehensive similarity measures and bi-random walk algorithm.  *Bioinformatics*, 32(17):2664–2671, 2016.

[25] Noël Malod-Dognin, Julia Petschnigg, et al. Towards a data-integrated cell. *Nature Communications*, 10(1):1–13, 2019.

[26] Ingrid A Mayer and Carlos L Arteaga. The pi3k/akt pathway as a target for cancer treatment.  *Annual Review of Medicine*, 67:11–28, 2016.

[27] Rachana Mehta and Keyur Rana. A review on matrix factorization techniques in recommender systems. In  *2017 2nd International Conference on Communication Systems, Computing and IT Applications (CSCITA)*, pages 269–274. IEEE, 2017.

[28] Nina Nikolova and Joanna Jaworska. Approaches to measure chemical similarity–a review.  *QSAR & Combinatorial Science*, 22(9-10):1006–1026, 2003.

[29] Byron P Roe, Hai-Jun Yang, and Ji Zhu. Boosted decision trees, a powerful event classifier. In  *Statistical Problems in Particle Physics, Astrophysics and Cosmology*, pages 139–142. World Scientific, 2006.

[30] Matthew Ruffalo, Mehmet Koyutürk, and Roded Sharan. Network-based integration of disparate omic data to identify “silent players” in cancer. *PLoS Computational Biology*, 11(12), 2015.

[31] Sudipa Saha Roy and Ratna K Vadlamudi. Role of estrogen receptor signaling in breast cancer metastasis. *International Journal of Breast Cancer*, 2012, 2012.

[32] Vıćctor Sánchez-Margalet and Souad Najib. Sam68 is a docking protein linking gap and pi3k in insulin receptor signaling. *Molecular and Cellular Endocrinology*, 183(1-2):113–121, 2001.

[33] Junrong Song, Wei Peng, and Feng Wang. A random walk-based method to identify driver genes by integrating the subcellular localization and variation frequency into bipartite graph. *BMC Bioinformatics*, 20(1):238, 2019.

[34] Amit Verma, Suman Kambhampati, et al. Jak family of kinases in cancer. *Cancer and Metastasis Reviews*, 22(4):423–434, 2003.

[35] David S Wishart, Yannick D Feunang, An C Guo, Elvis J Lo, Ana Marcu, Jason R Grant, Tanvir Sajed, Daniel Johnson, Carin Li, Zinat Sayeeda, et al. Drugbank 5.0: a major update to the drugbank database for 2018. *Nucleic Acids Research*, 46(D1):D1074–D1082, 2017.

[36] Guanming Wu, Xin Feng, and Lincoln Stein. A human functional protein interaction network and its application to cancer data analysis. *Genome Biology*, 11(5):R53, 2010.

[37] Jia-Ching Wu, Ching-Shu Lai, et al. Tetrahydrocurcumin, a major metabolite of curcumin, induced autophagic cell death through coordinative modulation of pi3k/akt-mtor and mapk signaling pathways in human leukemia hl-60 cells. *Molecular Nutrition & Food Research*, 55(11):1646–1654, 2011.

[38] Mengyun Yang, Huimin Luo, et al. Drug repositioning based on bounded nuclear norm regularization. *Bioinformatics*, 35(14):i455–i463, 2019.

[39] Hong-Nu Yu, Young-Rae Lee, et al. Induction of g1 phase arrest and apoptosis in mda-mb-231 breast cancer cells by troglitazone, a synthetic peroxisome proliferator-activated receptor $\gamma$ (ppar$\gamma$) ligand. *Cell Biology International*, 32(8):906–912, 2008.

[40] Yi Zeng and Nianyu Lai. Association between the cd28 c.17 +3 t¿c polymorphism (rs3116496) and cancer risk: An updated meta-analysis. *Medical Science Monitor*, 25:1917, 2019.

[41] Zhao Zhang, Jun Jiang, et al. Chimeric antigen receptor t cell targeting egfrviii for metastatic lung cancer therapy. *Frontiers of Medicine*, 13(1):57–68, 2019.

**Supplementary Figure Legends**


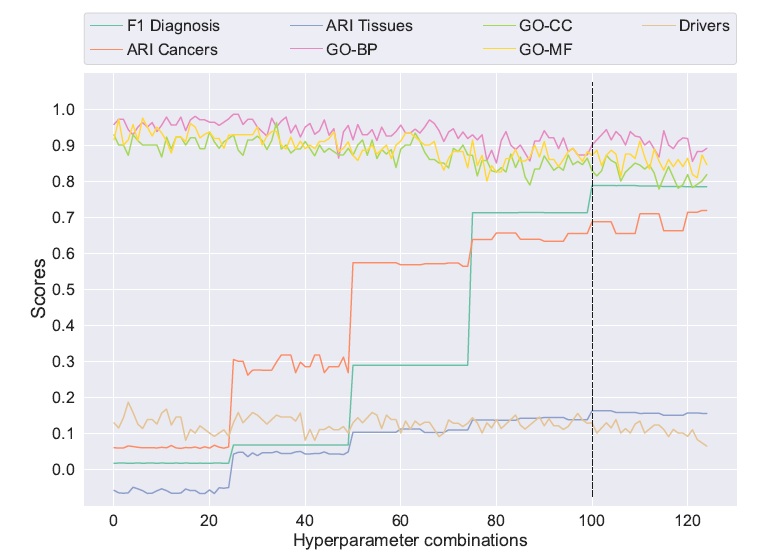


**Supplementary Fig. 1:** Framework sensitivity to hyperparameters with respect to different scores relating to patients and genes embeddings. Sequence of hyperparameter combinations is defined with set product $(k_{1},k_{2},k_{3}) \in\{2,5,10,15,21\} \times\{70,80,90,100,110\} \times\{40,50,60,70,80\}$. Black dashed vertical line indicates the best set of hyperparameters according to the macro-F1 score with respect to patients’ diagnosis.

**
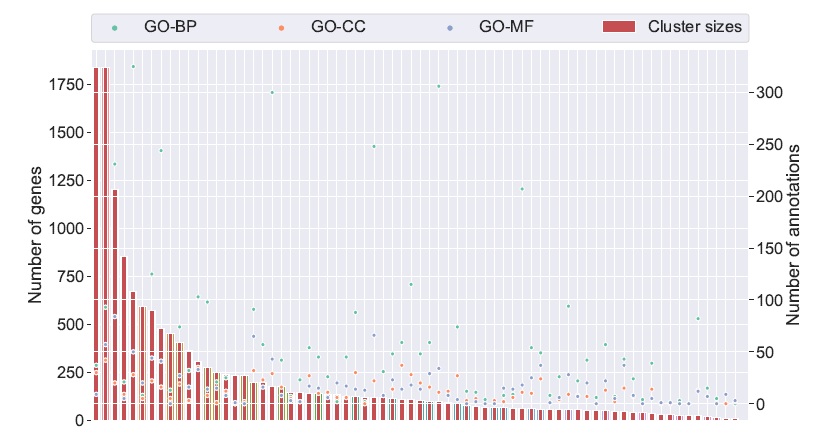
**

**Supplementary Fig. 2:** Distribution of gene cluster sizes and associated numbers of enriched annotations for each category of the GO ontology.


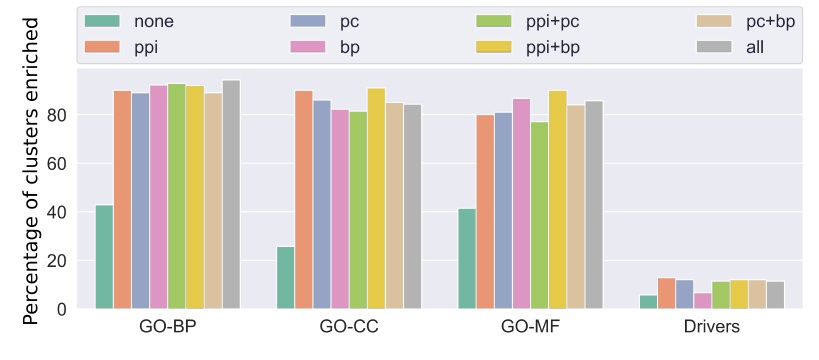


**Supplementary Fig. 3:** Evolution of enrichment scores based on gene data ablation.


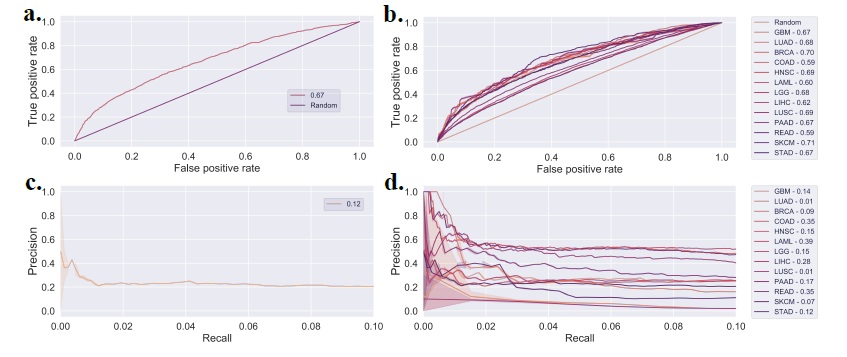


**Supplementary Fig. 4:** Global validation against CCGD driver genes of our predicted associations between genes and cancer types. Top row gives receiver operator curves for **a.** all predictions and **b.** per cancer type predictions. The bottom row give precision recall curves for **c.** all predictions together and **d.** per cancer type predictions. Precision-recall curve are cut at recall $0.1$ to show top ranked precision of predictions in more detail. Each value in the legends corresponds to either AUROC or AUPRC score of the non-restricted associated curve.


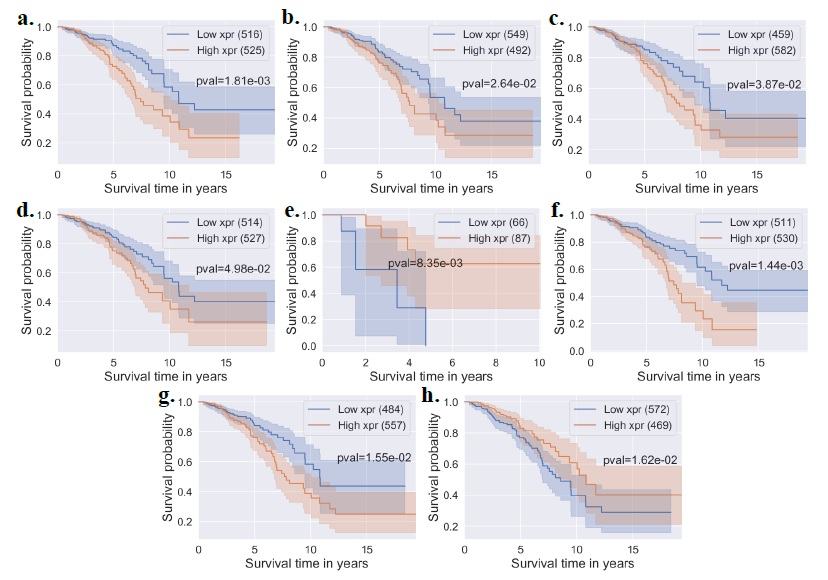


**Supplementary Fig. 5:** Kaplan-Meir curves comparing patients survival within a given cohort based on the relative expression of a gene. Each panel corresponds to a gene–cancer pair: **a.** KAT2B–BRCA, **b.** MDM2–BRCA, **c.** SP1–BRCA, **d.** HERC1–BRCA, **e.** HERC1–READ, **f.** NCOA3–BRCA, **g.** CHD6–BRCA, and **h.** PPARG–BRCA. Numbers in parenthesis in legends correspond to the numbers of patients falling in the associated category.


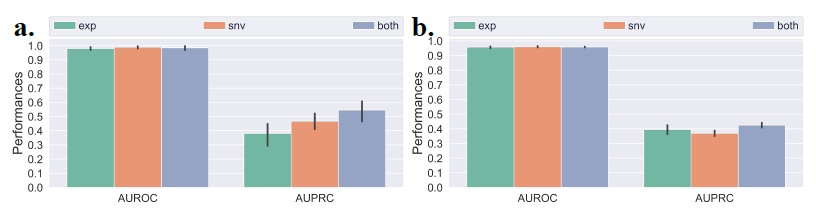


**Supplementary Fig. 6:** Ablation of patient–gene data effect on performances for **a.** drug–cancer type and **b.** gene–cancer type link prediction tasks.


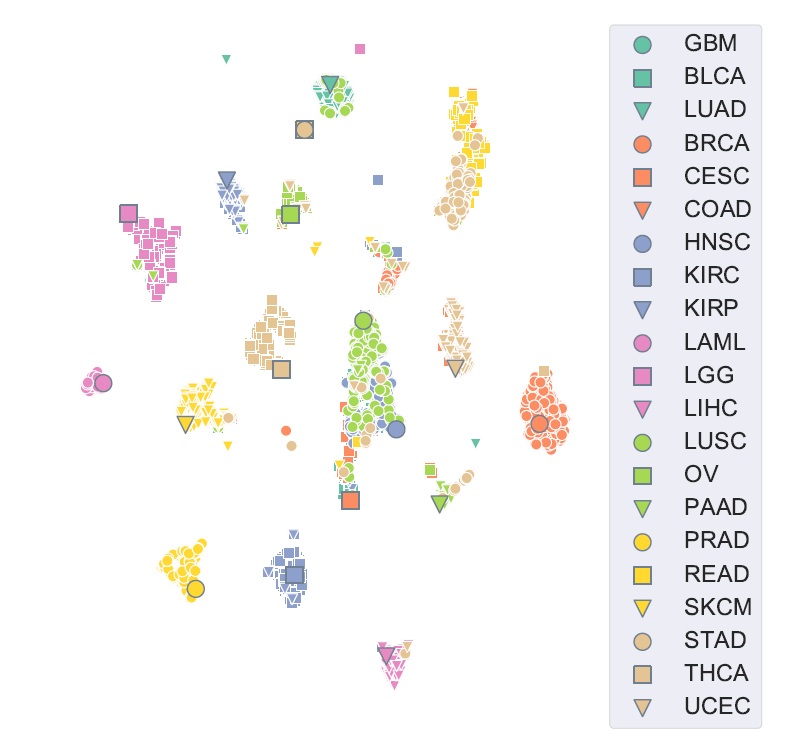


**Supplementary Fig. 7:** UMAP plot representing the embedding of patients and cancer types in the latent space. The larger circled markers correspond to the embeddings of cancer types, and the smaller ones represent the embeddings of patients. Colours and shapes indicate cancer types (see Supplementary Table 1 for abbreviations meanings).

**Supplementary Tables**

**Supplementary Table 1:** List of cancer types considered in this study with associated abbreviations from TCGA.

| Cancer | Cohort size | Abbreviation | Cancer | Cohort Size | Abbreviation |
| --- | --- | --- | --- | --- | --- |
| Acute Myeloid Leukemia | 173 | LAML | Bladder Urothelial Carcinoma | 295 | BLCA |
| Brain Lower Grade Glioma | 439 | LGG | Breast invasive carcinoma | 1,041 | BRCA |
| Cervical squamous cell carcinoma and endocervical adenocarcinoma | 259 | CESC | Colon adenocarcinoma | 428 | COAD |
| Glioblastoma multiforme | 159 | GBM | Head and Neck squamous cell carcinoma | 480 | HNSC |
| Kidney renal clear cell carcinoma | 518 | KIRC | Kidney renal papillary cell carcinoma | 222 | KIRP |
| Liver hepatocellular carcinoma | 173 | LIHC | Lung adenocarcinoma | 477 | LUAD |
| Lung squamous cell carcinoma | 428 | LUSC | Ovarian serous cystadenocarcinoma | 262 | OV |
| Pancreatic adenocarcinoma | 142 | PAAD | Prostate adenocarcinoma | 375 | PRAD |
| Rectum adenocarcinoma | 153 | READ | Skin Cutaneous Melanoma | 430 | SKCM |
| Stomach adenocarcinoma | 415 | STAD | Thyroid carcinoma | 500 | THCA |
| Uterine Corpus Endometrial Carcinoma | 508 | UCEC |  |  |  |

**Supplementary Table 2:** Details of the data used. The columns correspond to: 1) the type of data, 2) the size of the matrix representing the data, 3) the density, where applicable, indicates the percentage of the existing links between the entities out of all possible links, and 4) the symbol used in the document to refer to the matrix containing the corresponding data.

| Data | Size | Density | Symbol |
| --- | --- | --- | --- |
| Gene expression | 7,998$\times$15,224 | n.a | $X_{exp}$ |
| Gene SNV | 7,998$\times$15,224 | $1.7\%$ | $X_{snv}$ |
| Patient cancer type | 21$\times$7,998 | n.a. | $X_{pct}$ |
| PPI | 15,224$\times$15,224 | $0.21\%$ | $X_{ppi}$ |
| Protein complexes | 7,022$\times$15,224 | $0.05\%$ | $X_{pc}$ |
| Biological pathways | 1,650$\times$15,224 | $0.32\%$ | $X_{bp}$ |
| Drug–target | 7,333$\times$15,224 | $0.013\%$ | $X_{dt}$ |
| Drug tanimoto similarity | 7,333$\times$7,333 | n.a. | $X_{ts}$ |

**Supplementary Table 3:** GO annotations statistics for all GO subtypes.

| GO subtype | GO–BP | GO–MF | GO–CC |
| --- | --- | --- | --- |
| Number of annotations | 2,322 | 538 | 366 |
| Percentage of genes annotated | $50\%$ | $43\%$ | $45\%$ |

**Supplementary Table 4:** Link between cancer specific clustering and confounders (sex and age). Each cohort is separated in two clusters and we compute the ARI score with patient’s sex and the Mann-whitney U significance score between the two distributions of ages. The results show no significant link between clustering and confounders (except age for LGG). Note that CESC, OV, PRAD, and UCEC only affect one sex.

| Cancer | Sex (ARI) | Age (p-value Mann-whitney U test) |
| --- | --- | --- |
| All patients | 0.015 | 0.068 |
| GBM | −0.022 | 0.917 |
| BLCA | 0.0005 | 0.573 |
| LUAD | 0.008 | 0.204 |
| BRCA | −0.017 | 0.523 |
| CESC | 0 | 0.593 |
| COAD | −0.0005 | 0.23 |
| HNSC | −0.01 | 0.746 |
| KIRC | 0.009 | 0.27 |
| KIRP | −0.0195 | 0.522 |
| LAML | −0.00001 | 0.219 |
| LGG | 0.0005 | 0.01 |
| LIHC | −0.0002 | 0.36 |
| LUSC | −0.008 | 0.109 |
| OV | 0 | 0.77 |
| PAAD | 0.016 | 0.213 |
| PRAD | 0 | 0.473 |
| READ | 0.016 | 0.553 |
| SKCM | −0.01 | 0.233 |
| STAD | 0.011 | 0.495 |
| THCA | −0.005 | 0.4 |
| UCEC | 0 | 0.984 |

**Supplementary Table 5:** F1 scores measuring how closely new patients are embedded into the latent space to their cancer type and patients having the same cancer.

| Closest cancer macro-F1 | Nearest neighbours macro-F1 |
| --- | --- |
| $0.773\pm0.028$ | $0.882\pm0.015$ |

**Supplementary Table 6:** Top 10 protein complexes associated to cancer types.

| Protein Complex | Predicted for |
| --- | --- |
| IL6:sIL6R:IL6RB:JAKs | all cancers |
| p-7Y-RUNX1:PTPN11 | BRCA;BLCA;PAAD;LUSC;GBM |
| R-HSA-1112759 | BRCA;LAML;BLCA |
| Integrin alpha2bbeta3:SRC | BRCA;BLCA |
| R-HSA-1112753 | BRCA;LAML;BLCA;LGG;GBM |
| R-HSA-1112563 | BRCA |
| SAM68:p120GAP | BRCA |
| JAKs:OSMR | BRCA |
| IL6ST:JAKs | BRCA |
| R-HSA-9632399 | BRCA |
